# Quantifying the Resolution Ceiling of Prototype-Based Few-Shot Segmentation on Sub-Centimetre Pulmonary Nodules: A Training-Free Geometric Analysis

**DOI:** 10.3390/diagnostics16183027

**Published:** 2026-09-18

**Authors:** Joon-Ho Cho

**Affiliations:** Department of Electronics Convergence Engineering, Wonkwang University, 460 Iksandae-ro, Iksan 54538, Jeollabuk-do, Republic of Korea; cho1024@wku.ac.kr

**Keywords:** few-shot segmentation, pulmonary nodule, lung CT, feature resolution, Dice coefficient, upper bound, LIDC-IDRI, benchmark design, image preprocessing

## Abstract

**Background/Objectives:** Prototype-based few-shot segmentation is validated almost entirely on centimetre-scale abdominal organs; on pulmonary nodules, 70% of which are less than 10 mm in size, accuracy degrades sharply. We seek to determine how much of that degradation is geometric rather than methodological. **Methods:** No published method is trained or evaluated here. For any model whose prediction is a union of whole feature grid cells, the attainable Dice coefficient is bounded exactly by a single ratio ρ of the lesion diameter to the grid cell extent; bilinear upsampling relaxes it rather than removing it. We measured this bound on the 50% consensus masks of 2614 LIDC-IDRI nodules, quantified lesion preservation under six mask-handling policies, and swept a training-free implementation across fifteen input geometries. **Results:** Using SSL-ALPNet’s configuration, no whole-cell decision at that grid can exceed 19.0% Dice on the 5–8 mm band, which holds 44.6% of nodules, compared with 63–85% on abdominal organs. The governing size is the slice’s equal-area diameter, i.e., a median of 69% of the nominal value, which overstates the bound by 11.5 points. A lung mask used to erase rather than to locate destroys the lesion it should help find; hard masking removes more than half of 11.7% of the 180 nodules (95% CI 7.8–17.2). The swept implementation tracks the ceiling (r = 0.74 per episode), attaining 69% of the ceiling at the derived 32 mm tile compared with 8% at full-chest input; at a fixed cell size, it peaks at the encoder’s training resolution. **Conclusions:** Much of the reported deficit is bounded by representation geometry rather than by modelling quality. We provide a lesion-preserving formulation and derive the tile size from measurement rather than via a chosen margin.

## 1. Introduction

Annotation scarcity is the practical ceiling on deep learning for medical image segmentation, and few-shot segmentation is the field’s most direct answer to this problem. Given a handful of annotated support images, the task is to segment a query image containing the same structure. Within this family, prototypical networks dominate; a class prototype is formed by masked average pooling over support features, and query pixels are labelled by their similarity to the prototype. Self-supervised variants removed the need for annotated meta-training altogether, and the combination of superpixel pseudo-labels with adaptive local prototype pooling in SSL-ALPNet [1] remains the reference point. Subsequent work refined the prototype representation [2,3,4], substituted a vision foundation model for the encoder [5], and adapted promptable segmenters.

What these methods share is their evaluation set. Synapse abdominal CT, CHAOS abdominal MRI, and MS-CMRSeg cardiac MRI account for essentially all reported results; a recent review and comparative study of few-shot medical segmentation evaluates only abdominal organs and kidney tumours [6]. The targets in these benchmarks—the liver, spleen, kidneys, ventricles—are roughly convex and measure centimetres to tens of centimetres across. Pulmonary nodules exhibit neither of these characteristics. Across the 2614 nodules referenced in the LIDC-IDRI, the median diameter is 7.8 mm, and 70.1% measure less than 10 mm; margins may be spiculated or lobulated, and ground-glass lesions differ from surrounding parenchyma by only tens of Hounsfield units.

The imaging field has noted this difficulty, but only in passing. Q-Net observes that “the SOTA ADNet performs poorly for small-sized organs” [2]. SSL-ALPNet motivates local prototypes by noting that extreme foreground–background imbalance causes a class to be “spatially averaged into a 1-D representation prototype” [1]. A retrieval-augmented method reports Dice coefficients of 0.22–0.34 on cardiac regions below 200 pixels against 0.81–0.88 above this level [7]. Outside the few-shot setting, the sensitivity of overlap metrics to discretisation at small object sizes is documented as a validation pitfall [8,9]. What is missing is a quantitative account of the mechanism and the degree of the deficit explained by any particular cause.

The one prior application of this method family to lung lesions, MSPO-Net [10], compares PANet, ALPNet, ADNet, and Q-Net on a private CT cohort of tuberculosis, aspergillosis, and adenocarcinoma. It reports mean intersection-over-union (IoU) only, does not stratify by lesion size, and targets centimetre-scale consolidations rather than sub-centimetre nodules. It notes, without quantifying, that “the lesion area of lung adenocarcinoma is usually small, which only can provide limited features for foreground prototype learning.”

This paper supplies the missing account. Our contributions are as follows.

**A measured resolution ceiling.** For any method that makes its decision on a downsampled feature grid and upsamples that labelling, e.g., the prototype family studied here, as opposed to architectures with a learned decoder that recovers sub-cell detail (Section 4.4), the attainable Dice is bounded by a function of a single dimensionless quantity: the ratio ρ between lesion diameter and the physical extent of one feature grid cell. The bound is exact for predictions that are unions of whole cells; Section 4.4 and Section 4.6 explain the effect of bilinear upsampling and learned decoders on this bound. We measure this bound directly on the 50% consensus masks of 2614 LIDC-IDRI nodules rather than deriving it from an idealised shape, and we show that it is already binding at the input configurations published for these methods. Using SSL-ALPNet’s own settings, no whole-cell decision at that grid can exceed 19.0% Dice on the 5–8 mm band that holds 44.6% of all nodules, compared with the 63–85% level reported for the same method on abdominal organs.

**A correction regarding how lesion size is stated.** The size that governs a two-dimensional model’s performance is not the nominal three-dimensional diameter that annotations record, but the equal-area diameter of the slice the model actually sees, which is a median of 69% of the nominal diameter. Reasoning from the nominal figure overstates the ceiling by 11.5 percentage points. Once size is measured this way, real nodule outlines behave like discs of equal area to within one point—the deficit is one of area, not shape.

**A lesion-preserving region formulation and evidence that it is necessary.** Restricting the search to the lung is the cheapest way to reduce ρ, but a lung mask used to erase rather than to locate discards part of the lesion it was meant to help find. Across 180 nodules, hard masking destroys more than half of 11.7% of them; a learned mask reduces this result to 2.2% but does not eliminate the destruction and moves one nodule in the opposite direction. Dilating the mask before cropping, or cropping without erasing, leaves every lesion intact.

**A measurement of how much of the bound binds.** A bound that nothing approaches, and whose ordering nothing follows, would be a curiosity. Swept across nine input geometries with no training of any kind, a reference implementation of the same prototype rule tracks the ceiling (r = 0.74 per episode, 0.98 across configuration means) and attains 69% of it at the tile we derive, compared with 8% at a full-chest input. What remains of the gap is the widest at the exact point at which a coarse grid also starves the support prototype—a second cost of the same cell size, which the bound does not charge for. This is a controlled measurement of geometry, not an evaluation of any published system: SSL-ALPNet, ADNet and Q-Net are named throughout as the configurations whose input geometry at which we read the bound, and none of them are trained or run in this study. The fraction of the bound recovered by a trained method is the subject of the companion study.

**A public, reproducible protocol.** Unlike the only prior lung evaluation of this method family, every measurement here is reproducible from public data, and the code and derived tables are released at https://doi.org/10.5281/zenodo.21721043.

### Related Work

**Prototype-based few-shot medical image segmentation.** PANet [11] introduced prototype alignment for few-shot segmentation. In the medical domain, SSL-ALPNet combined superpixel-based self-supervised pseudo-labels with adaptive local prototype pooling, removing the need for annotated meta-training and establishing the evaluation protocol still used in the field. ADNet [12] replaces the similarity threshold with a learned anomaly score; Q-Net [2] adds query-informed refinement; DSPNet self-refines prototypes toward high-fidelity detail [3]; RPT introduces a region-enhanced prototypical transformer [4]. Ayzenberg et al. replaced the encoder with DINOv2-large and adapted it with low-rank adaptation (LoRA) [5]. All are evaluated on Synapse, CHAOS, and MS-CMRSeg.

A recent comparative review reimplements SSL-ALPNet and evaluates it on both abdominal organs and kidney tumours under an identical pipeline [6]. On organs, it reports Dice coefficients of 52.1 to 74.2; on one-shot kidney tumour segmentation, the same implementation reports results of 23.9, and conventionally trained ALP-Net achieves levels of 30.5. The authors attribute the drop to a mismatch between tumours and the superpixel structure the self-supervision relies on, noting that tumours “can appear as small, irregular segments within an organ”. The drop is thus documented, in an independent implementation, without being attributed to scale.

Two protocol distinctions from SSL-ALPNet are relevant. Setting 1 permits test classes to appear as the background in training images; Setting 2 removes such images entirely, so test classes are genuinely unseen. Because self-supervised pre-training consumes whole images, Setting 1 leaves the encoder implicitly exposed to the test class, and the reported Dice is correspondingly optimistic.

**Few-shot methods on lung lesions.** MSPO-Net [10] is, to our knowledge, the only prototype-based few-shot evaluation performed on lung lesions. It compares PANet, ALPNet, ADNet and Q-Net on a private cohort of 202 CT cases covering secondary tuberculosis, pulmonary aspergillosis and lung adenocarcinoma, reporting a mean IoU between 0.71 and 0.87 across the four baselines and three diseases, at an effective stride of 8 on a 417-pixel crop. It does not stratify by lesion size, and its targets are centimetre-scale consolidations rather than sub-centimetre nodules. One detail of its results is relevant to the present work: across the three diseases, lung adenocarcinoma is the only one for which a baseline outperforms the proposed method, and of it the authors write that the “lesion area is usually small, which only can provide limited features for foreground prototype learning”. The observation is qualitative and is not pursued.

An active-contour few-shot framework for lung nodules has also been reported [13]. It is not episodic meta-learning and uses no prototypes; “few-shot” there denotes training on few patients rather than a support-query protocol. It is relevant here because its final segmentation is produced by a variational model evolving at pixel resolution on a lung-region crop, and it reports Dice near 0.90 on LIDC. A method whose decision is not taken on a coarse lattice is not subject to the bound derived below, which we take up in Section 4.4. No work in this family has been evaluated on sub-centimetre pulmonary nodules, and none has reported accuracy stratified across clinically meaningful diameter bands.

Object scale, feature resolution, and overlap metrics. That downsampled representations bound achievable overlap is an established form of argument. Achievable Segmentation Accuracy was introduced to measure the ceiling imposed by superpixel granularity [14], and upper-bound analyses of representations have been treated as a research object in their own right [15]. Marin et al. [16] derived a boundary-error bound of O(√(D/N)) for uniform sampling. For detection, Wang et al., motivating a normalised Gaussian Wasserstein distance for tiny-object detection, showed that IoU for a 6 × 6 object collapses from 0.53 to 0.06 under a one-pixel shift, against 0.90 to 0.65 for a 36 × 36 object [8]. Reinke et al. catalogue this as a validation pitfall [9]. In the vision transformer (ViT) literature, patch size has been tied empirically to tumour volume [17], and feature upsampling has been proposed precisely because vision transformers incur a significant resolution reduction [18]. None derives a Dice ceiling as a function of object-to-cell ratio, and none applies the argument to few-shot segmentation. Four differences separate the present bound from that lineage, and we state them here because the general form of the argument is not new. Achievable Segmentation Accuracy bounds a partition into irregular superpixels, whereas the grid whose cells bound a feature-map decision is regular and its cell size is fixed by the network and the crop through Equation (1). The measure bounded there is pixel accuracy or a boundary error, not the Dice coefficient these methods report. The bound is evaluated here stratified by the clinical diameter band rather than pooled. And the quantity we ask for is not the bound itself but the fraction of it a system attains, which is what separates a geometric shortfall from a modelling one.

**Lung field segmentation as preprocessing.** Lung field segmentation on CT is effectively saturated on non-pathological anatomy: the reported Dice is 0.98–0.99, and a controlled comparison of four architectures found differences below 0.02, concluding that performance is “primarily a data diversity problem, not a methodology problem” [19]. Failures concentrate on consolidation, effusion, atelectasis and masses, where threshold-based and learned methods alike exclude tissue that is at soft-tissue density. We therefore treat lung segmentation as preprocessing rather than as a contribution, and focus in Section 2.5 and Section 3.3 on how the mask is used rather than on how it is produced.

**Nodule subtype annotation in LIDC-IDRI.** LIDC-IDRI provides, for each nodule marked ≥3 mm, nine characteristic ratings alongside the contour. The texture rating on a 1–5 scale is conventionally mapped to ground-glass (1–2), part-solid (3), and solid (4–5), a mapping used in nodule texture classification [20] and in the construction of LNDb [21]. We adopt this mapping. Note that internalStructure and calcification are nominal rather than ordinal, and cannot be averaged across readers.

Table 1 sets these configurations beside one another in the terms that matter for the bound. Two methods that differ in backbone and in every architectural detail may sit in the same geometric regime, and two runs of one architecture may sit in regimes an order of magnitude apart; neither fact is visible from the input resolution alone. The table also records where the bound applies exactly, where it is relaxed, and where it does not apply. It is deliberately short. Rows are restricted to methods whose backbone and input size are both stated in the source, and even for those the physical field of view of the input crop usually has to be assumed rather than read—which is the reporting gap Section 4.5 asks the field to close.

## 2. Materials and Methods

**Figure 1:**
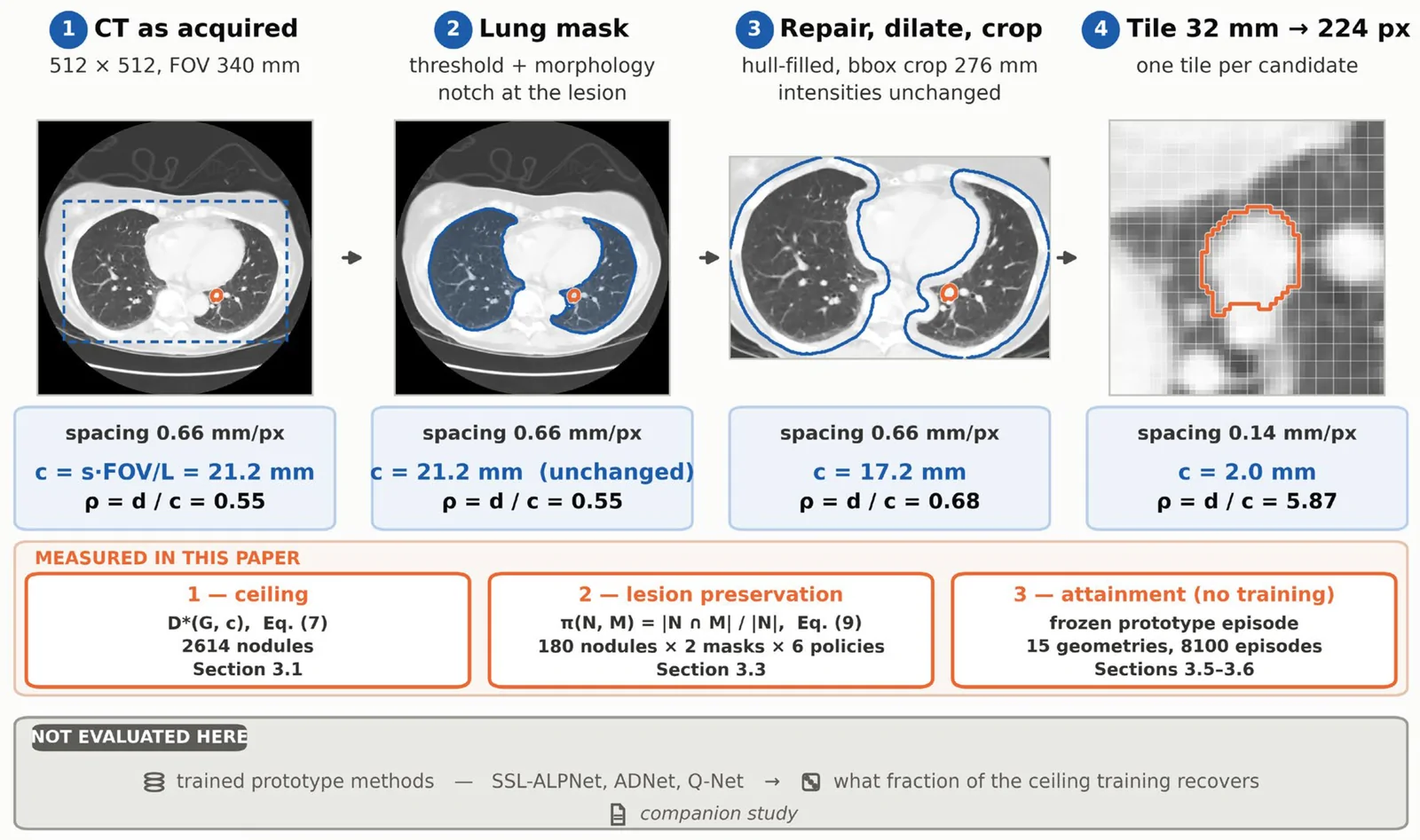
The chain, and where the feature cell size goes. One axial slice of LIDC-IDRI-0045, in four numbered stages: (**1**) the CT as acquired, (**2**) the threshold lung mask, (**3**) the hull-repaired mask cropped to its bounding box without altering intensities, and (**4**) the 32 mm tile of Section 3.4. The series shown has a 340 mm field of view, so its cell sizes sit slightly below the 21.9 mm the text quotes for a nominal 350 mm chest. Orange is the expert contour, blue the retained region; the mask carries a notch at the lesion, which is why it must locate and not erase. The band beneath the chain names the three measurements this paper reports; the grey band below it is what the paper does not measure.

This section states the two quantities the paper measures, the pipeline in which they are measured, and the procedure by which the tile size is chosen. Figure 1 places each of them in the imaging chain. The results are reported in Section 3.

### 2.1. Data and Reference Masks

We used LIDC-IDRI, a public collection of 1018 thoracic CT scans from 1010 patients assembled by seven academic centres and eight medical imaging companies and distributed by The Cancer Imaging Archive (TCIA), Version 4 (updated 21 September 2020), https://doi.org/10.7937/K9/TCIA.2015.LO9QL9SX, accessed on 9 July 2026, under a Creative Commons Attribution 3.0 licence [22,23,24]. Nodules were annotated in a two-phase reading: four experienced thoracic radiologists first marked lesions independently and blinded to one another, then reviewed their own marks together with the anonymised marks of the other three, contouring every nodule judged to be 3 mm or larger and rating nine semantic characteristics [22]. TCIA distributed the collection de-identified; no attempt at re-identification was made, and this study was a secondary analysis that generated no new patient data. Contours and ratings were read with pylidc 0.2.3 [25], which distributed them with the package, so every measurement in Section 3.1 and Section 3.2 is reproducible without downloading any pixel data.

Annotations were grouped into nodules by the package’s clustering routine, and the reference mask of a nodule was the 50% consensus of the available contours, the convention in segmentation work on this database. We recorded the number of contributing readers per nodule, because the consensus is not uniformly well determined: 33.9% of nodules were contoured by all four readers, 17.9% by three, 18.5% by two and 29.2% by one. Two sampling grids were used, and which applies where matters at this object size. The ceiling measurements of Section 3.1 and Section 3.2 and the truncation measurement of Section 3.4 were performed on consensus masks resampled to 1 mm isotropic spacing, so that a cell size in millimetres equals a cell size in pixels. The preservation measurements of Section 3.3 were made at the acquisition sampling grid of each series, because the lung mask and the nodule contour were both defined there and resampling either would introduce interpolation into a ratio of areas; Section 3.3 reported π on that grid. The attainment sweep of Section 3.5 resampled each tile to the network input size, as Section 2.7 describes. For display only, every CT panel in this article was processed identically—total-variation denoising (weight 0.010) followed by an unsharp mask (σ = 1.0, amount 0.40)—and is shown in the same lung window (level −600 HU, width 1500 HU). No quantity reported here was computed from the displayed pixels.

**Figure 2:**
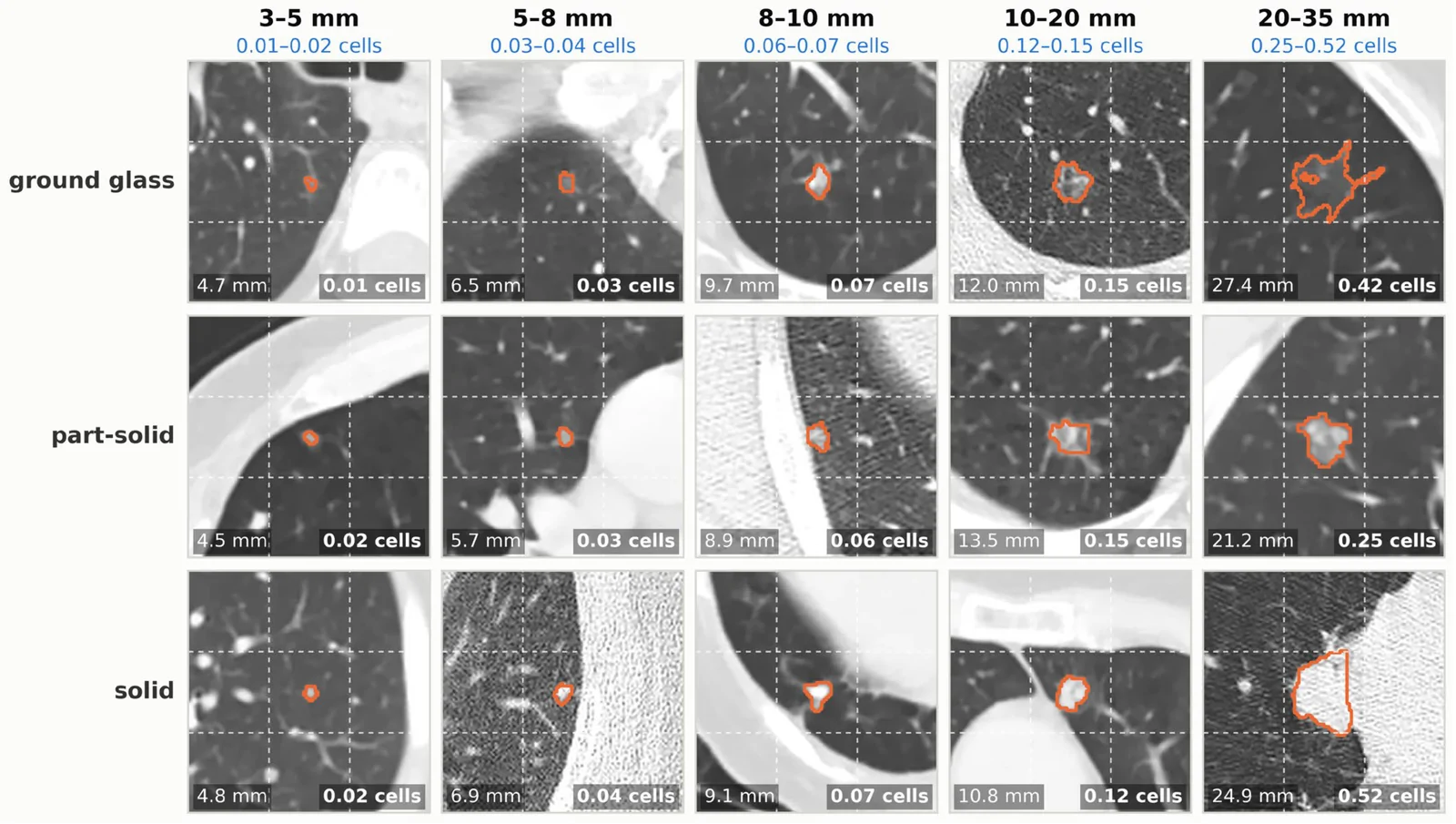
The benchmark, and the grid a 224-pixel chest input puts on it. Fifteen nodules, one per diameter band and subtype, each selected as the median slice-equivalent diameter of that cell—by size alone, with no performance consulted. Every panel shows the same 65.7 mm of anatomy and the same 3 × 3 lattice of 21.9 mm cells, which is what a ViT-B/14 at 224 pixels produces on a 350 mm field of view. Orange is the 50% consensus contour; the value at the left of each panel is the nominal diameter and the value at the right is how many lattice cells the lesion covers, taken as the area of a disk of the slice-equivalent diameter divided by the area of one cell. The largest coverage among the fifteen is 0.52 cells, a 25 mm solid mass; none reaches a single cell.

Each nodule carries two sizes. The nominal diameter is the mean of the readers’ reported three-dimensional diameters and defines the bands 3–5, 5–8, 8–10, 10–20 and 20–35 mm; the slice-equivalent diameter of Equation (3) is what enters ρ. Restricting to nodules with a nominal diameter between 3 and 35 mm gave 2614 nodules from 863 patients, of which 83.9% were solid, 10.9% were ground-glass and 5.2% were part-solid; the median nominal diameter was 7.78 mm, and 70.1% fell below 10 mm. Figure 2 shows one nodule from each band and subtype on the feature grid that a 224-pixel full-chest input produces.

The two measurements that require pixel data—lesion preservation in Section 3.3 and the attainment sweep in Section 3.5—used a 30-series subset selected before any measurement to populate all fifteen subtype-by-band cells, of which 28 series yielded nodules in the bands after consensus, giving 180 nodules. The selection was deterministic and is reproducible from the released code. Series with a slice thickness above 2.5 mm were excluded first. The remaining series were then added one at a time, each step taking the series that maximises Σ w(s)/(1 + *n*(b, s)) per gigabyte of CT, where the sum runs over the distinct (band, subtype) cells that series contributes, *n*(b, s) is how many nodules the already-selected series place in that cell, and w is a rarity weight of 3 for part-solid, 2 for ground-glass and 1 for solid nodules, roughly in inverse proportion to their prevalence in the corpus. The division by *n* makes the first example of a cell worth far more than the fifth, and the division by size keeps the download tractable; neither term consults any measurement made later. The resulting inventory—patient, nodule index, subtype, nominal and slice-equivalent diameter, band and reader count—has been released, so the subset can also be reproduced as a list. The truncation measurement of Section 3.4 needs only the consensus masks and was therefore made on all 2614.

Because two of the measurements were made on this subset rather than on the full corpus, its relation to the corpus should be stated. The selection maximises coverage of the fifteen subtype-by-band cells rather than reproducing their prevalence, so the subset is deliberately not proportional: part-solid nodules are 20.0% of it against 5.2% of the corpus, ground-glass 28.3% against 10.9%, and the two extreme diameter bands are likewise over-represented—3–5 mm at 12.2% against 7.8% and 20–35 mm at 10.0% against 7.7%—while the most populous 5–8 mm band falls from 44.6% to 33.3%. This is the intended consequence of the rarity weighting, and it has one implication for reading the results: a rate pooled over the subset is not an estimate of the corpus rate, and we have therefore reported the preservation and attainment results stratified by band and by subtype rather than pooled alone. Within a band, the subset is close to the corpus in the quantity that matters for the ceiling: the median slice-equivalent diameter differs by 0.27, 0.27, 0.47, 0.39 and 0.76 mm in the five bands, in every case a small fraction of the band width. The subset is thus representative of size within a stratum and deliberately unrepresentative of how often each stratum occurs.

### 2.2. The Feature-Cell Size and ρ

Let a model produce its decision on a feature grid whose cells correspond to c × c millimetres of the input, and let the prediction be upsampled to input resolution. For a convolutional backbone, c is the output stride times the pixel spacing; for a vision transformer, it is the patch size times the pixel spacing. Because CT is resized to a fixed input size, c depends on the field of view of the crop as well as on the network,

(1) $$c=\frac{s\cdot FOV}{L}$$

where *s* is patch size or stride in pixels, FOV is the physical extent of the input crop in millimetres, and *L* is the input side length in pixels. Equation (1) makes explicit that *c* is determined by the crop, not by the scanner; cropping to a smaller field of view at fixed *L* reduces c proportionally. This is the lever we use in Section 2.4.

Define the dimensionless ratio as

(2) $$\rho =\frac{d}{c}$$

where d is the lesion diameter. Section 3.2 shows that d must be the equal-area diameter of the slice the model is presented with,

(3) $$d_{slice}=2\sqrt{\frac{A}{\pi }}$$

with A the foreground area of the consensus mask on that slice, rather than the nominal three-dimensional diameter the annotation records.

Four quantities recur and are not interchangeable, so we fix the names here. The nominal diameter *d_nom* is the reader-averaged three-dimensional diameter the annotation records, and it defines the diameter bands used for stratification throughout. The slice-equivalent diameter *d_slice* of Equation (3) is the equal-area diameter of the single axial slice a two-dimensional model is presented with, a median 69% of *d_nom*, and it is the one that enters ρ. The feature-cell size *c* of Equation (1) is the physical extent of one cell of the grid on which the decision is taken, in millimetres, and it is set by the crop as much as by the network. The ratio ρ = *d_slice*/c is dimensionless and is the single variable the ceiling depends on. Where a band is named in millimetres it is always a band of d_nom; where a ceiling is quoted it is always a function of ρ.

### 2.3. The Oracle Bound

Let *G* ⊂ ℝ^2^ be the lesion region on one axial slice and let {*C_k*}, k = 1 … K, be the cells of the feature grid, each of area c^2^. A model that decides at grid resolution and upsamples by nearest assignment produces a prediction that is a union of whole cells,

(4) $$P\left(S\right)={⋃}_{k\in S}C_{k},\;\;S\subseteq \{1,\;\ldots ,\;K\}$$

and its accuracy is the Dice coefficient

(5) $$Dice\left(P,G\right)=\frac{2\;\left|P\cap G\right|}{\left|P\right|+\left|G\right|}$$

We ask for the best such prediction. Writing a_k = |C_k ∩ G| for the overlap of cell k with the lesion, the oracle is

(6) $$D^{*}\left(G,c\right)={max}_{S\subseteq \{1,\;\ldots ,\;K\}}\;Dice\left(P\left(S\right),G\right)$$

This maximum has a closed form. For any S with |S| = m the denominator of (5) equals |G| + m c^2^, which depends on S only through m, so among subsets of size m the maximiser is the m cells of largest overlap. Ordering the overlaps decreasingly as a_(1)_ ≥ a_(2)_ ≥ … and writing A_m = Σ_{k ≤ m} a_(k)_ for their partial sums,

(7) $$D^{*}\left(G,c\right)={max}_{1\leq m\leq K}\;\frac{2A_{m}}{\left|G\right|+mc^{2}}$$

and the maximum is attained at finite m because a_(k)_ vanishes for cells disjointed from G. Equation (7) is exact, not a heuristic; it is what any classifier in this grid is choosing among, and no feature extractor, prototype construction, or similarity function can exceed it within the whole-cell decision model of Equation (4). We therefore call D* the whole-cell grid-resolution ceiling wherever the distinction matters. An implementation that upsamples a soft score map bilinearly, or that carries a learned decoder, decides on a finer effective partition than Equation (4) allows, and is subject to a correspondingly looser bound; Section 4.4 and Section 4.6 give the size of that relaxation, measured on the sweep of Section 3.5.

The quantity still depends on where the grid falls relative to the lesion. Writing D*(G, c; τ) for the oracle under a sub-cell translation τ of the grid, we report the average over a deterministic *n* × *n* lattice of offsets,

(8) $${\bar{D}}^{*}\left(G,c\right)=n^{-2}\sum_{i,j}D^{*}\left(G,c;\tau_{ij}\right),\;\;\tau_{ij}=\left(\frac{ic}{n},\;\frac{jc}{n}\right)$$

with *n* = 6. The determinism matters, and the reason is not the one an intuition about skew would give. Averaging over a random sample of offsets is unbiased for the same quantity; what it is not is precise. Enumerating every integer offset at each cell size on all 2614 masks gives the exact offset-averaged value, and against it the 6 × 6 lattice is within 0.34 points at every cell size we use, whereas a single randomly drawn offset carries a standard deviation of up to 3.9 points and individual draws that miss by as much as 8.8 points. Thirty-six random draws still leave errors of up to 2.1 points. What is biased rather than merely noisy is the practice of taking one grid aligned to the image origin: that estimate is 6.1 points high at c = 8 mm and 5.0 points high at c = 11 mm, but 2.6 points low at c = 4 mm and 1.6 points low at c = 2 mm, so the error cannot be corrected by a constant. Enumeration costs n^2^ evaluations of a closed form and removes both problems, which is why n = 6 is used throughout and why the offsets are enumerated rather than sampled.

**Figure 3:**
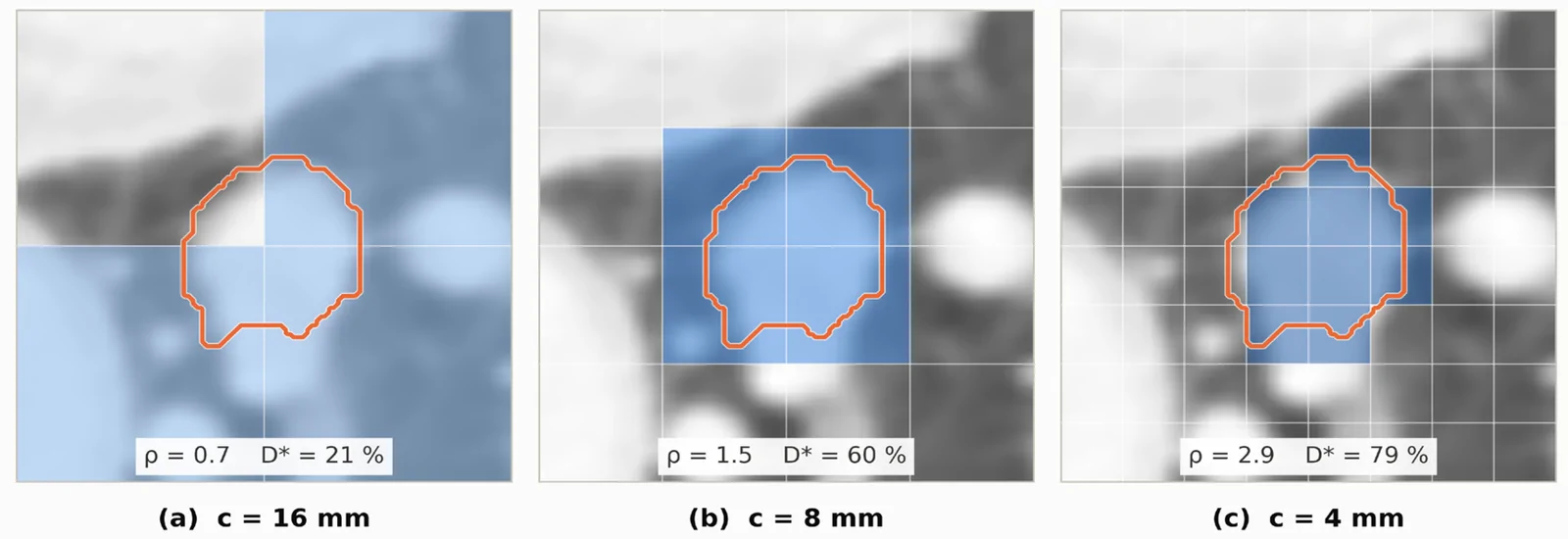
What Equation (7) selects, on the CT. The nodule of Figure 1—13.9 mm nominal, 11.7 mm across the slice shown—in a 32 mm window on three feature grids: (**a**) c = 16 mm, (**b**) c = 8 mm, (**c**) c = 4 mm. Blue is the subset of whole cells maximising Dice against the 50% consensus contour in orange, which is the best prediction any classifier deciding on that grid could produce. Values are for this single grid offset, whereas Table 2 averages over the offset lattice of Equation (8).

We evaluate (7) and (8) on real masks rather than on an assumed shape. For each nodule we take the 50% consensus mask of its largest axial slice, resampled to 1 mm isotropic spacing so that a cell size in pixels equals a cell size in millimetres, and pool it into c × c cells to obtain the overlaps a_k directly. Figure 3 shows, on the CT, the cells that Equation (7) selects at three cell sizes.

**Table 2:** Oracle Dice (%) by feature-cell size and nominal diameter band.

c (mm)	3–5 mm	5–8 mm	8–10 mm	10–20 mm	20–35 mm
**2** ^a^	**69.2**	**79.6**	**85.4**	**89.6**	**93.9**
3	55.1	68.6	76.6	83.5	90.5
4	42.4	59.2	69.2	78.0	87.2
6	25.3	42.0	56.3	68.3	81.2
**7** ^b^	21.4	37.8	50.2	62.8	78.2
8	17.2	29.1	43.7	59.5	75.6
10	10.3	21.4	35.0	51.7	70.5
**11** ^c^	9.4	**19.0**	30.1	46.6	68.2
14	6.0	13.2	21.9	37.6	61.4
18	3.8	8.1	14.5	28.0	53.0
22 ^d^	2.7	5.6	10.0	21.0	45.2

Measured on the 50% consensus masks of 2614 LIDC-IDRI nodules from 863 patients. Superscripts mark the cell size at which a published configuration operates. ^a^ The 32 mm region-of-interest tile derived in Section 3.4, at L = 224 and s = 14. ^b^ DINOv2-ALPNet, ViT-L/14, tested at 672 pixels. ^c^ SSL-ALPNet, ResNet-101 at output stride 8, 256-pixel input. ^d^ ViT-B/14 at a 224-pixel full-chest input. Cell sizes for the three published configurations assume a 350 mm chest field of view. Dice and the Jaccard index are deterministically related by J = D/(2 − D), so every value in this table can be read as an intersection-over-union without further measurement: the 19.0% Dice at c = 11 mm is an IoU of 10.5%. Bold marks the derived 32 mm tile (c = 2 mm), the cell sizes at which the published configurations operate, and the 19.0% value quoted in the Abstract and in Section 1.

### 2.4. Lesion-Preserving Region Extraction

Equation (1) shows that c falls linearly with the field of view of the crop. Restricting the crop to the lung and then tiling it is therefore the cheapest available lever; no architectural change, no additional parameters, and an order-of-magnitude reduction in c. With the 32 mm tile that Section 3.4 derives, at L = 224 and s = 14, c = 2.0 mm, which by Table 2 raises the ceiling on the 5–8 mm band from 19.0% to 79.6%.

Cropping to the lung alone is not enough. The chest field of view is roughly 350 mm and the two lungs together span about 280 mm, so cropping to the lung bounding box reduces c only from 21.9 mm to about 17.5 mm. Tiling is what moves c. The series of Figure 1 has a 340 mm field of view rather than the nominal 350 mm, so there the same two steps take c from 21.2 mm to about 17.2 mm, and then to 2.0 mm.

We obtain the lung mask with a pretrained public model rather than training one, for the reasons given in Section Related Work. Lung segmentation here is preprocessing, not a contribution.

Given a CT volume, we resample to 1 mm isotropic spacing, obtain a lung mask with a pretrained model, repair mask defects with morphological closing followed by convex-hull filling, dilate by δ mm, and crop to the bounding box of the repaired mask without altering voxel intensities. The extracted region is partitioned into overlapping tiles of physical side T mm, each resampled to the network input size, giving c = s·T/L. Support and query tiles are drawn under the same tiling, so prototypes are computed at the scale at which they are applied. Tile-level predictions are stitched with overlap averaging. Section 2.6 sets out the procedure that fixes T, and the preservation measurements of Section 3.3 fix δ.

The essential property is that the mask never removes information. It bounds the search region, which is what reduces c and therefore raises the ceiling; it does not decide what is lesion.

### 2.5. Mask-Handling Policies and the Preservation Ratio

**Figure 4:**
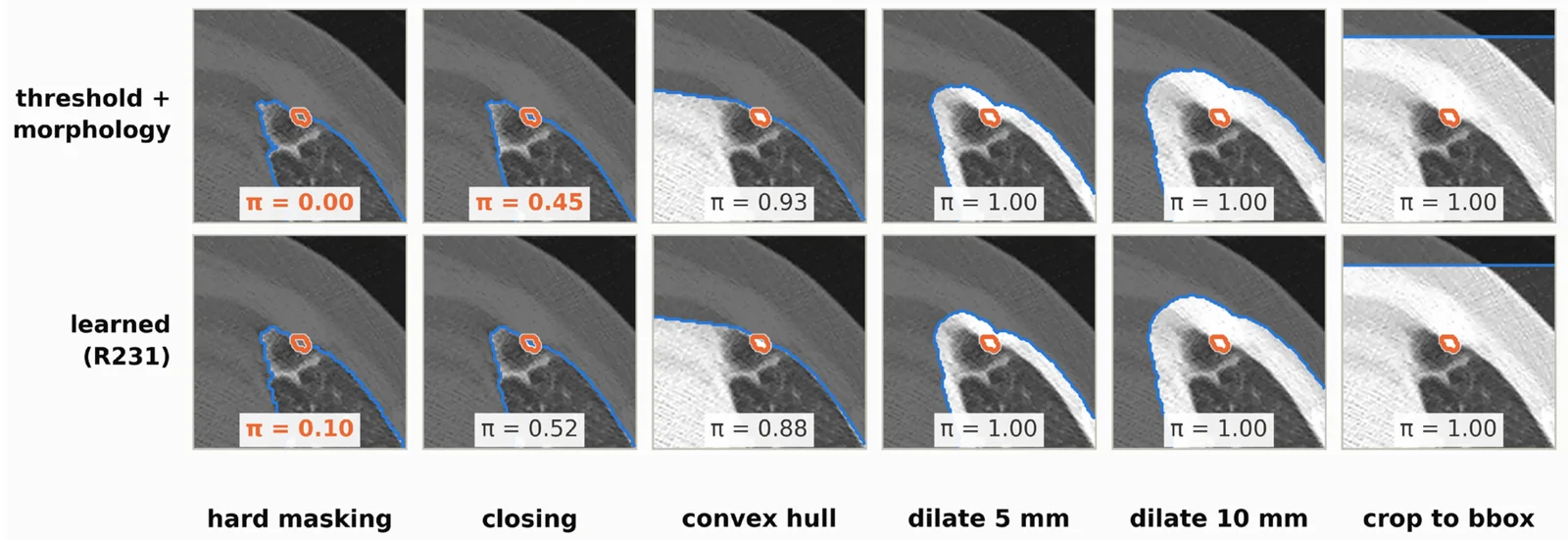
The six mask-handling policies, on a lesion where the choice matters. LIDC-IDRI-0466, a 6.7 mm nodule at the pleural surface, in the same 65.7 mm window throughout; the two rows differ only in how the lung mask was produced. Blue outlines what each policy retains, orange is the expert contour, and π is Equation (9), computed on the acquisition sampling grid so that the printed values are the ones behind the preservation rates of Section 3.3. Hard masking keeps none of this lesion (π = 0.00) and a learned mask a tenth (π = 0.10), while dilating or cropping without erasing keeps all of it.

The obvious way to use a lung mask is to zero everything outside it. This destroys part of what the pipeline exists to find. To quantify the effect we took 180 nodules from 28 patients, computed the lung mask two ways—threshold plus morphology, and the learned R231 model released by Hofmanninger et al. [19]—and measured, for each nodule, the fraction of its consensus mask surviving under six treatments of the same lung mask. Writing N for the nodule’s consensus mask and M for the region the treatment retains, the preservation ratio is

(9) $$\pi \left(N,M\right)=\frac{\left|N\cap M\right|}{\left|N\right|}$$

and we call a nodule severely truncated when π < 0.5, that is, when more than half of the evidence a segmenter would need has been removed before it is asked to segment. Equation (9) is deliberately generous to the mask; it counts a lesion as preserved whenever a bare majority of it survives. The six treatments are hard masking, which keeps the mask as produced; morphological closing; convex-hull filling; dilation by 5, 10 and 15 mm; and cropping to the bounding box of the dilated mask without altering voxel intensities. Figure 4 shows what each does to a lesion at the pleural surface, which is where the failure concentrates. The half is a reporting convention and not a clinical threshold, and we adopt it for two reasons rather than by calibration against outcome. It is the point at which the majority of the evidence is gone, so a segmenter presented with the remainder is being asked a different question from the one the annotation answers; and it is the level at which the outcome stops depending on where the cut falls, which a smaller criterion does not give. No clinical study establishes a preservation ratio below which a nodule is missed, and we do not claim one. Because the choice is a convention, Section 3.3 reports the whole distribution of π rather than the rate alone, together with the 10% criterion, so that a reader who prefers a different cut can read the corresponding rate off the figure.

### 2.6. Selecting the Tile Size

Reducing c is the lever, but by how much? The obvious answer—make the tile large enough to hold the largest lesion and no larger—needs a margin factor that has no principled value, and a margin chosen by intuition is an assumption presented as a derivation. Both sides of the trade-off can instead be measured.

The upper side is the ceiling, read backwards. Equations (7) and (8) give $\bar{D}$* as a function of ρ, so inverting them gives the ρ that a target implies, and Equation (1) turns it into a tile size,

(10) $$\rho^{*}={\bar{D}}^{*-1}\left(D_{target}\right),\;\;c^{*}=\frac{d_{q}}{\rho^{*}},\;\;T^{*}=\frac{c^{*}L}{s}$$

where d_q is the q-th percentile of the slice-equivalent diameter—the smallest lesions the target is required to hold for. The inversion is tabulated in Section 3.4, where the measured lower side is also given.

The two free choices in Equation (10) are the target ceiling D and the quantile q, and both should be argued rather than assumed. We take q = 25 because the design question in lung CT is not what the average nodule needs but what the small ones need: three quarters of LIDC nodules are larger than the 25th-percentile slice diameter of 4.07 mm and are therefore guaranteed at least the target, while the quartile below it is the population a screening programme exists to catch, and is where the curve is steepest, so a quantile much lower would drive the tile size down without a corresponding gain for the rest. We take D = 0.70–0.75 because that is the range in which the ceiling stops being the binding constraint: at a measured attainment of roughly two thirds of the bound, a 70% ceiling leaves a system able in principle to reach the high forties, which is the region where differences between methods become informative rather than being swamped by quantisation. Neither number is a clinical threshold, and we do not present them as one; they are stated here so that a reader who prefers different values can read the corresponding tile size straight off the ladder of Section 3.4.

### 2.7. A Reference Implementation for Measuring Attainment

A bound is worth what it predicts. To test whether this one binds in practice, we swept a reference implementation across the same range of cell sizes, choosing deliberately the simplest system that is still recognisably the method under study.

No training of any kind takes place. A frozen DINOv2 [26] ViT-B/14 encodes the tile; the support mask is pooled onto the token grid; the foreground prototype is the mean of the tokens the mask covers and the background prototype the mean of those it does not; the query score is the difference of the two cosine similarities, bilinearly upsampled to the input resolution and thresholded. This is the inference time mechanism of ALPNet with the learned parts removed. Removing them is the point: whatever the sweep shows cannot be attributed to a training recipe, an augmentation policy, or a choice of loss, because there is none.

Two details keep the coarse configurations from being measured unfairly. When no token is more than half covered—which at c = 21.9 mm is every episode—the single token of greatest overlap is taken as foreground, mirroring ALPNet’s guarantee of at least one prototype for objects smaller than the pooling window; without it the coarsest settings would produce no prediction at all and would drop out of the comparison rather than score badly in it. And Dice is reported at the best of forty thresholds per episode rather than at a fixed one, so that a shortfall cannot be an artefact of calibration. The forty thresholds are quantiles of that episode’s own score distribution, equally spaced from the 50th to the 99.9th percentile, so the grid is a description of the score range rather than a tuned parameter; the value it returns is an upper envelope over thresholds and is bounded above by the exhaustive optimum. Both choices move the measurement upward, which is the direction that makes the remaining gap to the ceiling harder to dismiss; Section 3.5 therefore reports, beside the oracle-threshold value, the Dice obtained at a single threshold held fixed across every episode of a configuration, which is the number a deployable system would face. Two properties of this threshold should be stated plainly, because they bound how the measured column may be read. It is chosen per episode, and it is chosen by scoring the candidate thresholds against the query mask itself—the same mask the episode is scored on. It is therefore an oracle quantity and is optimistic by construction: no deployed system can select a threshold this way, since the query annotation is what such a system is trying to produce. We use it deliberately, because the paper’s question is what the input geometry permits, and an oracle threshold removes calibration from the comparison so that the remaining differences are geometric. The deployable counterpart is the fixed-threshold column reported in Section 3.5, Section 3.6 and Section 3.7: one threshold chosen once and held across every episode of a configuration, which is what a real pipeline would set on a validation split and apply unchanged. The gap between the two columns is the size of the optimism, and it is not small—at the derived tile it is 56.6% against 36.2%. A deployment should therefore read the fixed-threshold column as its expected operating point and the oracle column as the geometric bound that column is approaching, and should calibrate the threshold on data held out from the cases it will be scored on.

Tiles are placed on the centroid of the consensus mask with a uniform random offset of up to ±8 mm on each axis, drawn once per nodule and reused by every configuration. The offset is not cosmetic. Centring exactly would fix the sub-cell grid alignment instead of sampling it—because L/2 is an exact multiple of the patch size at all three input sizes, an exactly centred lesion falls on the junction of four cells in every configuration, which is the least favourable of the alignments Equation (8) averages over—while the ceiling it is compared against is an average over alignments. Offsetting the centre samples the alignment instead, which both matches the definition of the ceiling and represents the localisation error any real candidate generator would carry. At ±8 mm, the offset spans a full cell width at every configuration but the coarsest, where it spans 73% of one; the residual bias this leaves in the ceiling is at most 3.4 points, at c = 10.9 mm, and under half a point at the coarsest.

The cohort is the 180 nodules from 28 patients of Section 2.5, spanning 2.22 to 23.1 mm of slice-equivalent diameter, and holding 22, 60, 37, 43 and 18 nodules in the five bands from smallest to largest—the outer two are thin, and per-band figures drawn from them are indicative rather than precise. Each nodule serves as the query three times, paired with a support nodule of the same subtype from a different patient, giving 540 episodes. The pairing is drawn once and reused by every configuration, so the settings differ in c and in nothing else, and the comparisons between them are paired. Nine combinations of tile size T and input size L place c between 21.9 and 2.0 mm by Equation (1), among them the full-chest ViT-B/14 input and the cell size of SSL-ALPNet’s published configuration, both marked in Table 2, and the 32 mm tile derived in Section 3.4. Two of the nine share c = 2.0 mm and differ only in tile size, which isolates the part of the outcome that the cell size does not determine. Section 3.6 reports two further sweeps over the same cohort and the same jitter offsets: two ladders that hold c fixed while the tile side and the input size move together, and a repeat of the nine configurations with the support drawn without regard to subtype.

### 2.8. Use of Generative AI Tools

Claude (Anthropic, San Francisco, CA, USA; Claude Opus 5, July–August 2026) was used during the preparation of this manuscript for drafting and editing the text, generating the analysis and figure code, and assistance with literature search and citation checking. No reported value originates from the tool itself; every quantity in Section 3 was computed by the archived code from the 50% consensus masks, and all AI-generated code was executed and its output inspected by the author. The author reviewed and edited all AI-assisted text and code, checked each figure, table and reference against the primary sources, and takes full responsibility for the content of this manuscript.

## 3. Results

### 3.1. The Ceiling by Cell Size and Diameter Band

The comparison to make is with the same methods’ reported abdominal results, which fall between 63% and 85% Dice. At its published settings, SSL-ALPNet cannot reach that range on the 5–8 mm band—which holds 44.6% of all LIDC nodules—no matter how good its features are, as long as the decision is taken at grid resolution; the ceiling is 19.0%. DINOv2-ALPNet, which tests at 672 and therefore operates at c ≈ 7 mm, reaches 37.8% on the same band. The gap is not evidence that prototypes fail on lung tissue; it is evidence that the grid is too coarse for the target. One qualification belongs with the 19.0% figure. SSL-ALPNet, like the reference implementation of Section 2.7, upsamples a soft similarity map bilinearly before thresholding, so 19.0% bounds the whole-cell decision its feature grid can express rather than the thresholded output that decision is turned into. Section 4.6 measures that relaxation on our own bilinearly upsampled sweep, where it lifts 6.2% of episodes above the offset-averaged whole-cell ceiling at an oracle threshold and 1.7% at a fixed one. The relaxation is real and it is small; it does not move the 19.0% figure into the 63–85% range that motivates the comparison.

**Figure 5:**
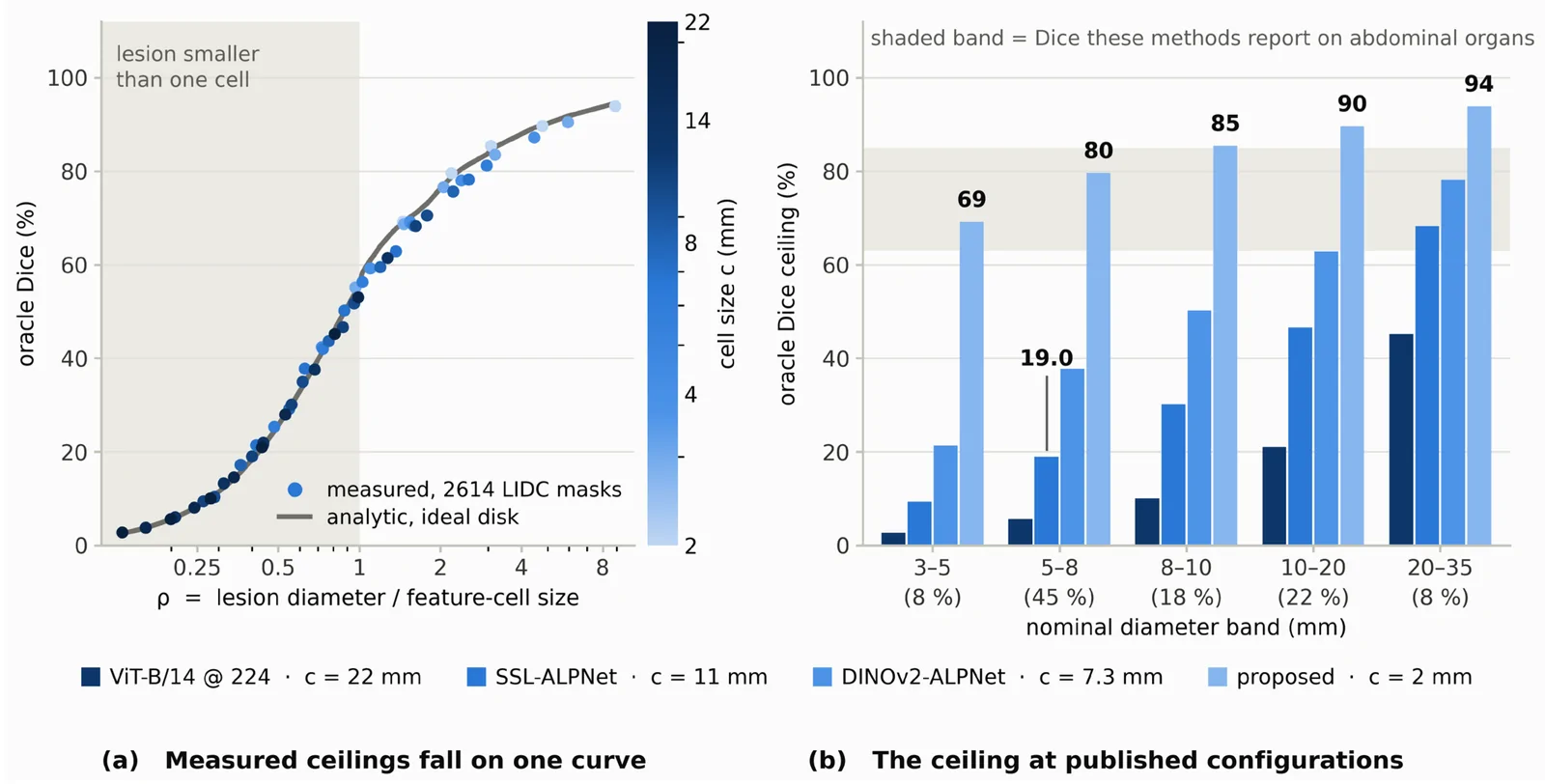
The resolution ceiling. (**a**) Measured oracle Dice for every combination of cell size and diameter band, plotted against ρ; the points fall on the analytic curve for an equal-area disk, which is the evidence that ρ, and not c and d separately, is the controlling variable. (**b**) The same ceiling read at the input configurations these methods are published with, against the 63–85% Dice they report on abdominal organs; the lightest bar of each group is the 32 mm tile derived in Section 3.4.

Figure 5 plots every (cell size, diameter band) pair against ρ. The points collapse onto a single curve—the 55 (cell size, band) means sit on the analytic equal-area-disk curve with R^2^ = 0.996 and a mean residual of 1.3 points—which is the evidence that ρ is the controlling variable rather than c and d separately.

Because the consensus is drawn from a single reader for 29.2% of nodules, the ceiling was recomputed stratified by reader count. Taken marginally, the strata differ; at c = 2 mm the mean ceiling is 77.5% for single-reader nodules against 87.9% for four-reader ones, and at c = 11 mm it is 20.3% against 40.5%. The difference is a size effect and not an annotation effect. Nodules a single reader marked are smaller—a mean slice-equivalent diameter of 4.82 mm against 8.87 mm for those all four marked—and ρ is what the ceiling depends on. Within matched slice-diameter bins the gap closes; at c = 4 mm it never exceeds 2.1 points in any bin from below 3 mm to above 14 mm, and averages 1.3 points, with the single-reader stratum marginally the lower throughout. Annotation uncertainty therefore shifts the level of the ceiling by about a point at matched size, and does not alter the relationship the paper reports.

### 3.2. Which Diameter Governs

Lesion size admits two definitions, and the distinction is decisive. Annotations record a nominal three-dimensional diameter. A two-dimensional model, however, is presented with one slice, whose foreground area corresponds to a smaller equivalent diameter. Across 2614 nodules the median ratio is 69.0%. Table 3 gives the ceiling each definition predicts, against the measured value.

Across all cell sizes and bands, the measured ceiling sits 1.0 point from the slice-area prediction and 11.5 points below the nominal one. Two consequences follow.

First, reasoning about attainable performance from the nominal diameter systematically overstates it, by more than eleven points at this cell size. Papers that report two-dimensional results while describing lesion size in nominal terms are comparing quantities that differ by a third.

**Figure 6:**
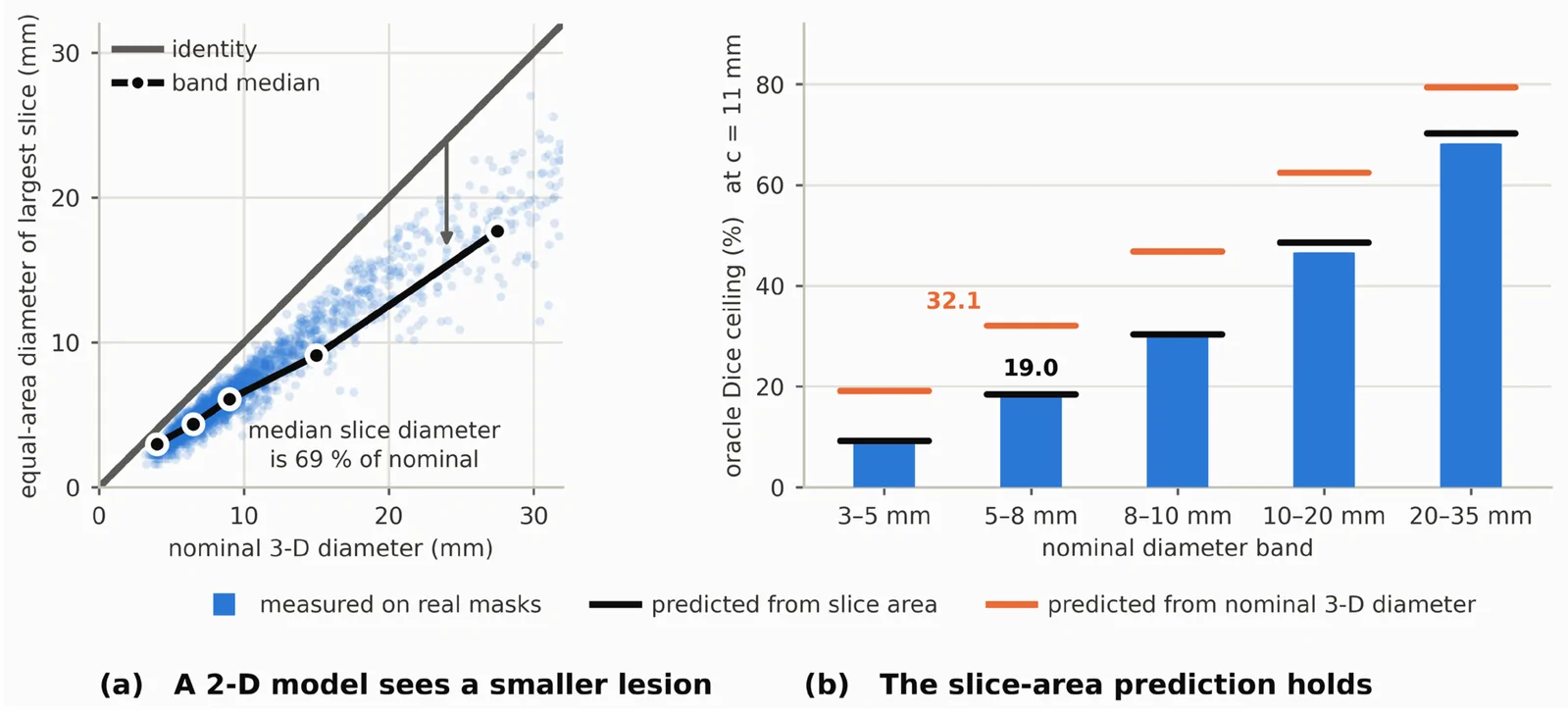
Nominal diameter overstates the lesion a two-dimensional model must segment. (**a**) Per nodule, the annotation’s nominal three-dimensional diameter against the equal-area diameter of its largest axial slice; the median ratio is 69%. (**b**) The ceiling predicted from each definition, against the measured value. The measurement tracks the slice-area prediction to within one point.

Second, real nodule outlines behave like disks of the same area. Spiculation and lobulation, which are the visually salient irregularities, do not measurably tighten the bound once area is matched. The deficit is area, not shape. Figure 6 shows both the systematic shrinkage from nominal to slice-equivalent diameter, and the fact that the slice-area prediction is the one that tracks the measurement.

### 3.3. Lesion Loss by Mask Quality and Policy

**Figure 7:**
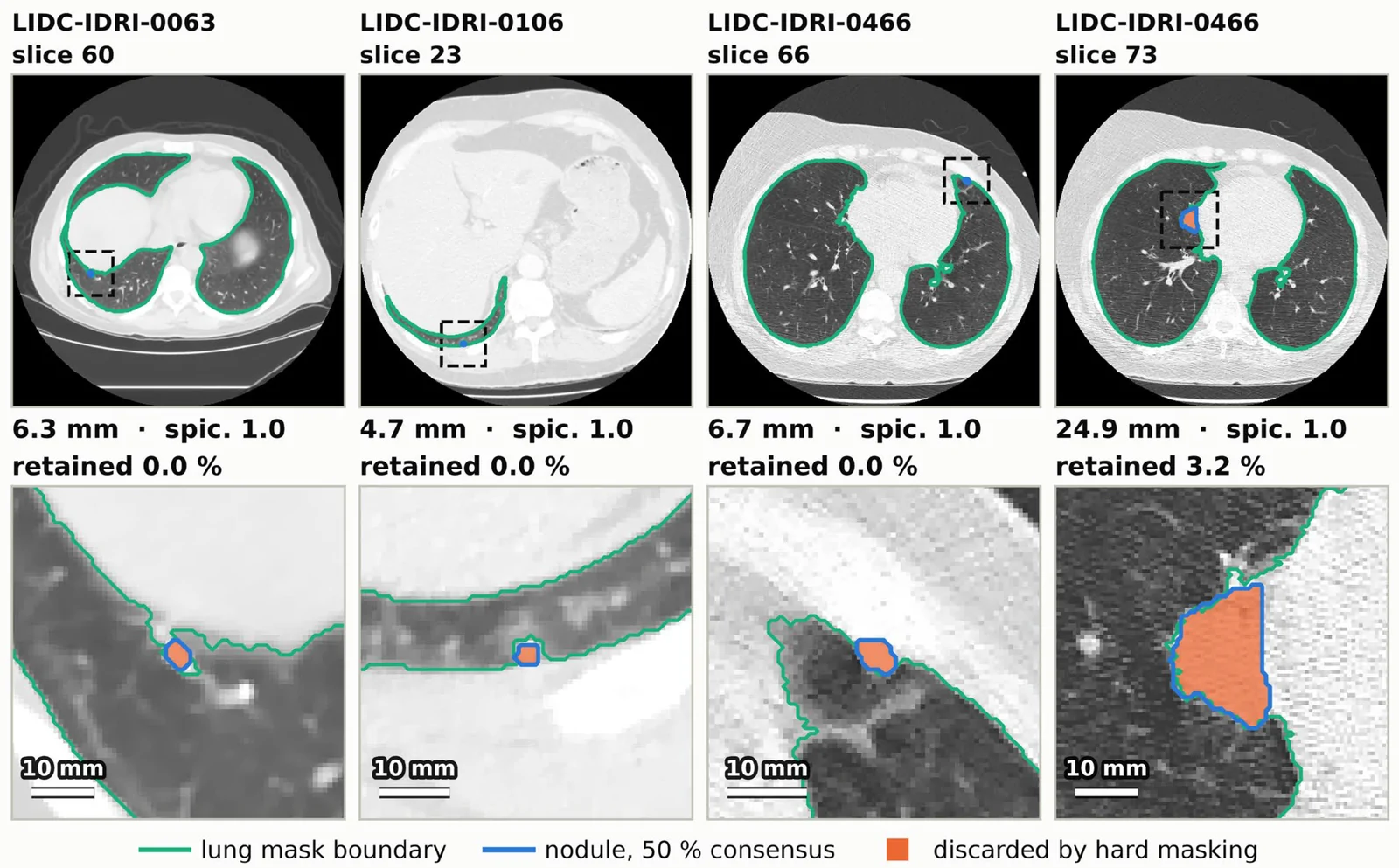
Using the lung mask to erase, rather than to locate, cuts into the lesion; the four worst hard-masking failures, on the CT. Top row, the full axial slice with the threshold lung mask boundary in green and the 50% consensus contour in blue, the dashed box marking the enlarged region; bottom row, that region enlarged, with orange marking the part of the lesion a hard mask discards. The window is identical in every panel (level −600 HU, width 1500 HU) and each scale bar is 10 mm. The two LIDC-IDRI-0466 panels are different nodules in the same series.

**Figure 8:**
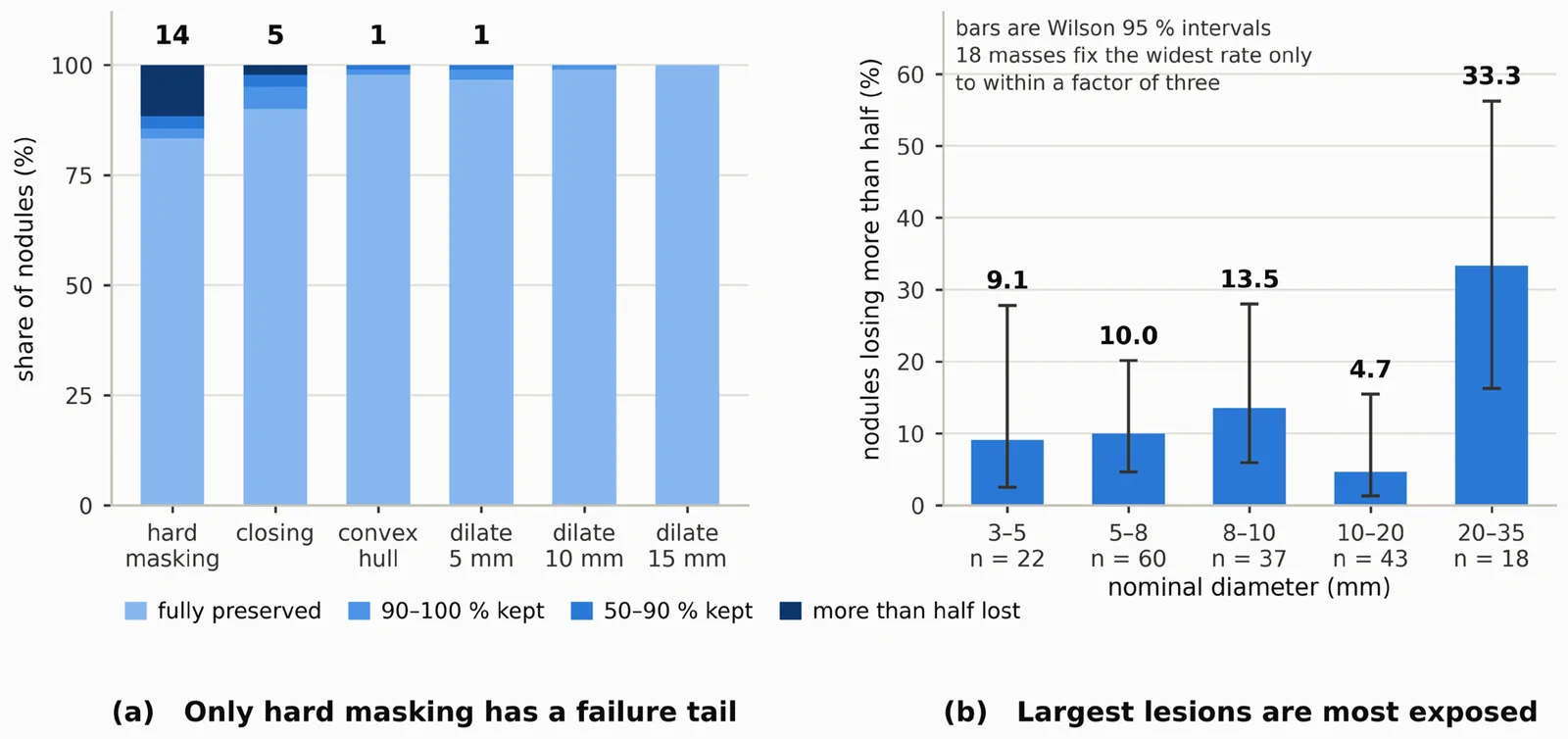
Lesion loss under the threshold-based mask. (**a**) Outcome distribution by masking policy; the number above each bar is the percentage of the 180 nodules that lose 10% or more, and only hard masking has a failure tail. (**b**) Nodules losing more than half, by nominal diameter band—the loss concentrates on the largest lesions, not the smallest. Bars in (**b**) are Wilson 95% intervals; the 20–35 mm band holds 18 masses, so its rate is fixed only to within a factor of three.

**Figure 9:**
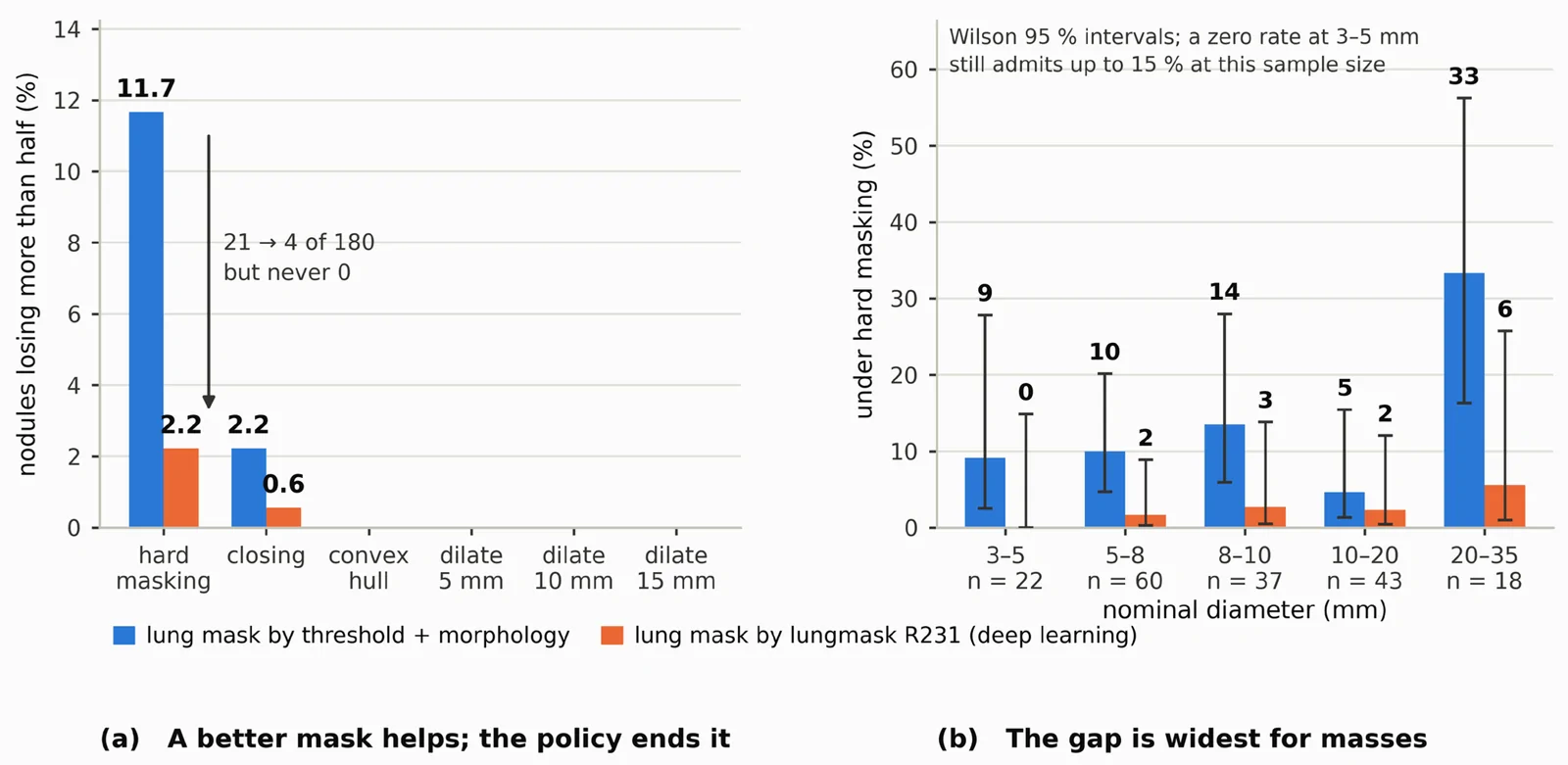
Mask quality against masking policy. (**a**) A learned lung mask cuts hard-masking failures from 11.7% to 2.2% but never to zero, whereas every policy from convex hull fill onward reaches zero with either mask. (**b**) The gap between the two masks is widest for masses above 20 mm. The bars in (**b**) are Wilson 95% intervals: at 3–5 mm the learned mask fails on no nodule, but at this sample size that is consistent with a rate as high as 15%.

Three findings follow, and Figure 7, Figure 8 and Figure 9 present them.

**Mask quality matters, and does not suffice.** Replacing the threshold mask with a learned one rescues 18 of the 21 nodules that hard masking had destroyed, cutting the failure rate from 11.7% to 2.2%. It does not reach zero. Four nodules still lose more than half; one 6.7 mm nodule retains 9.5% and one 24.9 mm mass retains 11.9%.

**The failures move as well as shrink.** One 10.8 mm solid nodule that the threshold mask preserved intact is cut to 41.4% by the learned mask. Improving the segmentation changes which lesions are destroyed as well as how many, so the failure mode is not reliably retired by a better model.

**The policy, not the mask, is what removes the loss.** Under every treatment from convex-hull fill onward, both masks preserve every lesion. Simply cropping to the dilated bounding box without altering voxel intensities preserves them by construction. This costs one morphological operation; the learned mask costs a deep model, its weights, and a GPU. Table 4 reports the rate under every policy, for both masks.

Figure 7 shows the four worst failures on the CT itself, and they fail in two distinct ways. Three are small nodules lying against a pleural or diaphragmatic surface, where the threshold mask boundary passes exactly around the lesion and keeps none of it: 6.3 mm in LIDC-IDRI-0063, 4.7 mm in LIDC-IDRI-0106 and 6.7 mm in LIDC-IDRI-0466. The fourth differs in kind—a 24.9 mm mass in that last series, at soft-tissue density against the mediastinum, which a threshold-based segmentation places outside the lung altogether and retains 3.2% of. What a learned mask then does to these four is the substance of the finding. It recovers the first two, keeping 100% and 76.5%, and fails on the other two, keeping 9.5% and 11.9%—both far below the half we call severe truncation. Mask quality helps, and it does not close the failure mode.

Stratifying by diameter reverses the intuition that small lesions are the fragile ones. Table 5 gives the rates by band.

Six of the eighteen masses above 20 mm in this subset—one in three, with a 95% Wilson interval running from 16% to 56%—lose more than half of their area to a threshold-based lung mask. The width of that interval should be read with the estimate: eighteen masses fix the rate only to within a factor of three, and the finding we would defend is that the failure exists and concentrates in this band, not its magnitude. The mechanism is straightforward; large, dense masses contact the chest wall or mediastinum and sit at soft-tissue density, so a threshold-based segmentation classifies them as outside the lung. This reproduces, on public data, the concentration of lung-segmentation failure on pathological tissue at soft-tissue density that was reported by Hofmanninger et al. [19]. It is also the band where the learned mask helps most, closing the gap from 33.3% to 5.6%.

This finding is clinically concerning; within this subset the failure concentrates on the largest lesions, which are those a reader is least willing to lose.

### 3.4. Truncation and the Resulting Range of Tile Sizes

Reading Equation (10) backwards gives the upper side of the range. Table 6 does so at the 25th-percentile slice diameter.

**Figure 10:**
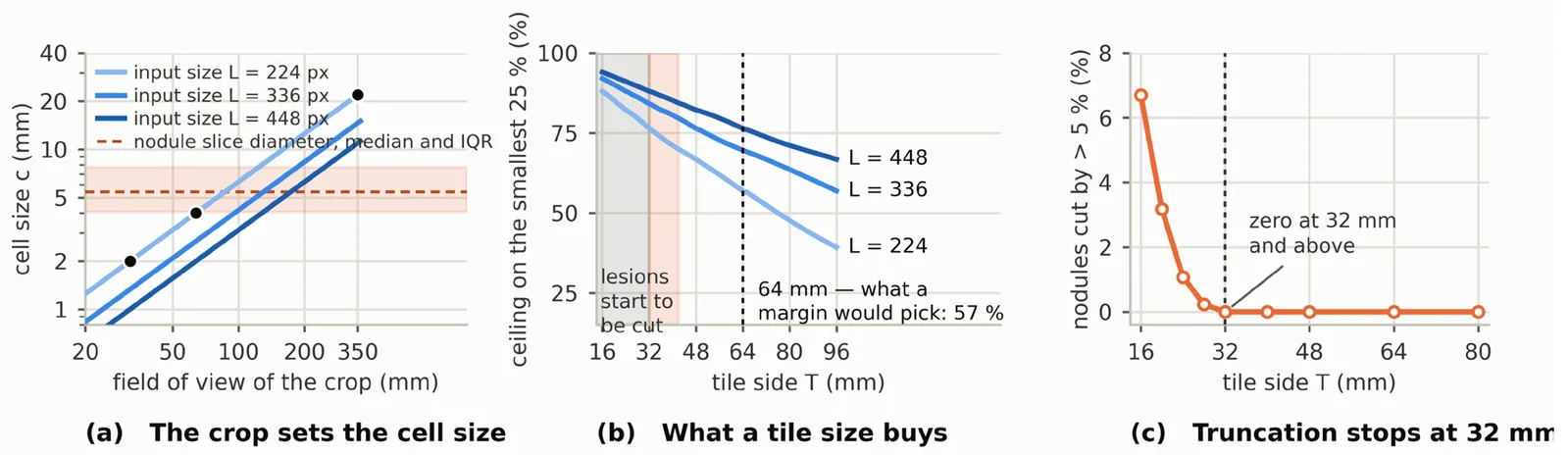
Choosing the tile geometry from geometry alone. (**a**) c is proportional to the field of view at fixed network and input size, so each configuration is a line and cropping moves along it; the band is the interquartile range of slice-equivalent diameters. (**b**) The ceiling a tile size buys on the smallest quartile of nodules, by Equation (10); the orange band is the 32–42 mm window in which both conditions hold, the grey band where lesions begin to be cut, and the dashed line that a factor-of-two margin would have picked. (**c**) The fraction of nodules losing more than 5% of their area to a centred tile, on all 2614 masks.

The procedure is therefore the following: Choose a target ceiling D and the quantile q of the lesion population for which it must hold; read ρ* from the measured curve; obtain c* = d_q/ρ* and T* = c* L/s. Then check the lower side: T must not be so small that lesions are cut, which the next paragraph measures rather than assumes. Any T between the truncation-free minimum and T* satisfies both, and the largest such T minimises the number of tiles. Figure 10a shows the design space that Equation (1) defines.

The lower side is truncation, and it is a measurement rather than a margin. For every nodule in the cohort we placed a square tile of side T on the centroid of the consensus mask and computed the fraction of the mask falling inside it. The result is sharper than the intuition it replaces: at T = 32 mm, the mean retained fraction is 99.99% and no nodule in the whole of LIDC-IDRI loses more than 5% of its area; at 24 mm, 1.1% of nodules do; at 16 mm, 6.7% do and seven lose more than half. Containment stops binding at 32 mm, not at the 60 to 70 mm that a factor-of-two margin on the largest mass would demand.

The two together bound the useful range with no model run. At L = 224, tiles between 32 and 42 mm reach a ceiling of 71 to 76% on the smallest quartile of nodules while truncating nothing; a 64 mm tile—the size that a factor-of-two margin on the largest mass would suggest—reaches only 57%, and is therefore larger than it needs to be. Raising the input to 336 moves the same window to 63–84 mm, which is the alternative if more physical context is wanted for other reasons. Figure 10 shows the three quantities together.

Two limits of the procedure should be stated. It says nothing about how small T may usefully become. Geometry alone favours ever smaller tiles, because the truncation penalty falls on the largest few per cent of nodules while the quantisation penalty falls on all of them; we verified that the composite of the two is monotone down to 16 mm. What must stop the shrinking is the localisation error of whatever supplies the candidate position, and that is not measured here. And the procedure optimises a bound, not performance. Section 3.5 measures how much of the bound one training-free implementation attains across the same range of cell sizes, and finds the derived tile to be the best of the nine configurations tried; whether trained methods recover more of the bound than frozen representations do is the subject of the companion study.

### 3.5. How Much of the Ceiling Is Attained

The reference implementation of Section 2.7 is training-free by design: the question here is whether the ceiling bounds and orders the output of a real system, not what a trained method scores—which the companion study takes up. Table 7 gives the reference implementation’s Dice at each of the nine configurations against the ceiling those same episodes imply—Equation (7) evaluated at each nodule’s own ρ = d_slice/c and averaged over the episode set, which is the estimator Section 3.1 licenses by showing the ceiling to be a function of ρ alone.

Three results follow, and they are separable.

**The bound is predictive.** Measured Dice rises with the ceiling at every step, from 1.1% at a full-chest 224-pixel input to 56.6% at the derived 32 mm tile—a factor of 50—while the encoder, the prototype rule, the episodes and the data are held fixed, and only the input geometry moves. Over all 4860 episodes, the correlation between a nodule’s ceiling and its measured Dice is r = 0.74 (Spearman 0.82); over the nine configuration means it is 0.98. A quantity computed from consensus masks with no model in the loop orders the outcomes of a model. On the 5–8 mm band, which holds 44.6% of LIDC nodules, the same progression runs from 2.5% measured against a 20.9% ceiling at SSL-ALPNet’s cell size to 52.7% against 77.1% at the derived tile.

**The bound is not tight, and it is loosest where Section 4.1 says it should be.** Attainment falls from 69.4% of the ceiling at c = 2.0 mm to 7.9% at c = 21.9 mm. This is the second consequence of a coarse grid, which starves the support prototype as well as quantising the output; at c = 21.9 mm the foreground prototype averages a single token in 100% of episodes, at c = 10.9 mm in 96%, and at c = 2.0 mm in 6%. Pooled across configurations, episodes whose prototype averages one cell reach 12.1% Dice, those averaging two or three reach 40.5%, and those averaging thirty or more reach 46.6%. The bound grants a perfect cell classifier; the measurement shows what the same grid does to that classifier’s input.

*The ceiling is necessary but not sufficient.* Two configurations share c = 2.0 mm—a 32 mm tile at 224 pixels and a 64 mm tile at 448—and therefore share a ceiling of 81.6%, yet they attain 69.4% and 54.2% of it. On the same 540 episode pairs the 32 mm tile is 12.5 points better (95% CI 10.7 to 14.2; better on 75% of pairs), the two differing only in how much of the tile is lung rather than lesion. Cell size fixes what is possible; the composition of the crop fixes how much of it is realised. A design procedure that reasons only about c, including the one in Section 2.6, is incomplete in a way this comparison makes visible, and Section 4.6 states it as a limitation rather than repairing it here.

A multivariable model separates these contributions rather than leaving them adjacent. Regressing per-episode Dice on the ceiling alone across all 4860 episodes, with standard errors clustered by patient, gives R^2^ = 0.540; a quantity computed from consensus masks with no model in the loop accounts for a little over half of the episode-level variance in what the model scores. Adding the number of foreground support tokens raises this to 0.547, and adding the logarithm of the tile side raises it to 0.570, at which point the support-token term is no longer distinguishable from zero (*p* = 0.67)—across these nine configurations a coarse cell and a wide tile arrive together, so the two are collinear here. Section 3.6 breaks that collinearity with two further ladders, and finds that the tile term is itself two terms. Lesion diameter and subtype add 0.6 points more, with part-solid nodules 4.1 points below solid at equal geometry (*p* = 0.02) and ground-glass indistinguishable from it (*p* = 0.62). Forty-two per cent of the episode-level variance is left unexplained by all of these together, and that residue is what a better encoder, a better prototype rule or a better calibration would have to win. The ceiling is thus the largest single term and not the only one, which is the reading Section 4.1 asks for.

Stratified reporting sharpens the same point. At the derived tile, mean Dice is 60.0% on solid nodules, 55.1% on ground-glass and 50.3% on part-solid, against band means rising monotonically from 42.1% at 3–5 mm to 67.1% at 20–35 mm; the subtype spread is smaller than the size spread, as the geometric account predicts, since subtype does not enter ρ. The medians run above the means throughout—50.0% against 42.1% in the smallest band—because the per-episode distribution has a floor at zero and no corresponding ceiling below the bound, so a mean is the conservative summary and is the one Table 7 reports. At the derived tile the mean of 56.6% sits below a median of 64.9%, with an interquartile range of 38.2 to 79.0%. The same skew is why the attained column of Table 7 is the ratio of the two column means rather than the mean of per-episode ratios; the latter runs lower at coarse cells because that distribution is strongly right-skewed—19.5% against 23.4% at c = 10.94 mm.

**Figure 11:**
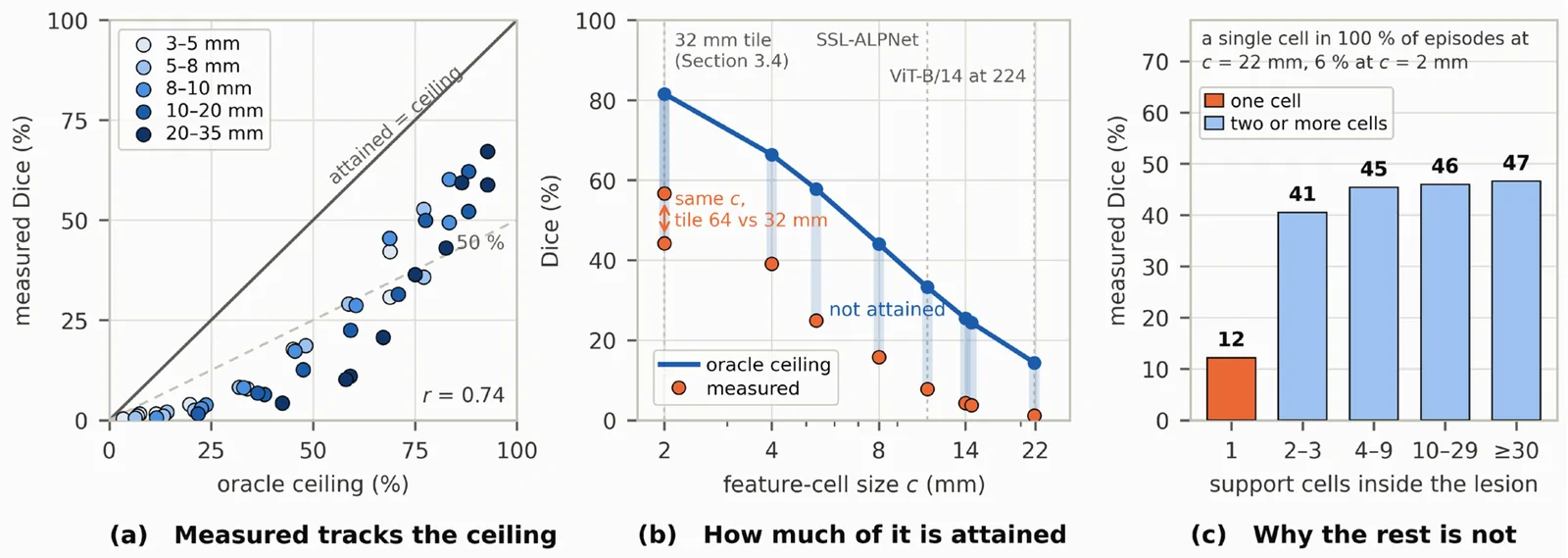
How much of the ceiling a training-free implementation attains. (**a**) Measured Dice against the ceiling, one point per configuration and diameter band, with the diagonal marking equality. (**b**) The same data against cell size; the shaded stems are the part of the ceiling not attained. (**c**) Dice against the number of feature cells the support mask covers, pooled over all 4860 episodes; the bars hold 3367, 481, 573, 325 and 114 episodes from left to right. Tile centres carry a random offset of up to ±8 mm, so the sub-cell grid alignment is sampled rather than fixed (Section 2.7).

Two subsidiary results are worth recording. The tile size derived in Section 3.4 from geometry alone is the best of the nine configurations measured, which is the only test of that derivation available without a trained system. And the advantage of an oracle threshold over a fixed one is 20.4 points at that configuration and 0.7 points at the coarsest; calibration is a second-order concern at fine grids and beside the point at coarse ones, where there is nothing left to calibrate. Figure 11 collects the attainment across configurations, cell sizes and support coverage.

### 3.6. Tile Size at Fixed Cell Size, and the Support Constraint

Two questions Section 3.5 left open can be settled with the same code and the same episodes. The first is what the tile size does at a fixed cell size; two configurations sharing c = 2.0 mm attain 69.4% and 54.2% of the same ceiling, but two points do not make a curve. The second is what the same-subtype pairing of Section 2.7 is worth. We therefore swept two further ladders, holding c fixed at 2.0 mm and at 4.0 mm while the tile side and the input size move together as L = 14 T/c, and repeated the nine published configurations with the support drawn from any other patient without regard to subtype. All three sweeps use the same 180 nodules and the same jitter offsets, and the ladders reuse the 540 episode pairs of Section 3.5, so every comparison below is paired. The two configurations common to Table 7 and Table 8 reproduce their earlier values exactly, episode by episode. One distinction is worth fixing before the numbers. Panel (b) of Figure 10 gives the ceiling a tile size buys, which is a statement about geometry alone and falls monotonically as the tile grows; what follows is the Dice a tile size achieves, which does not.

**Figure 12:**
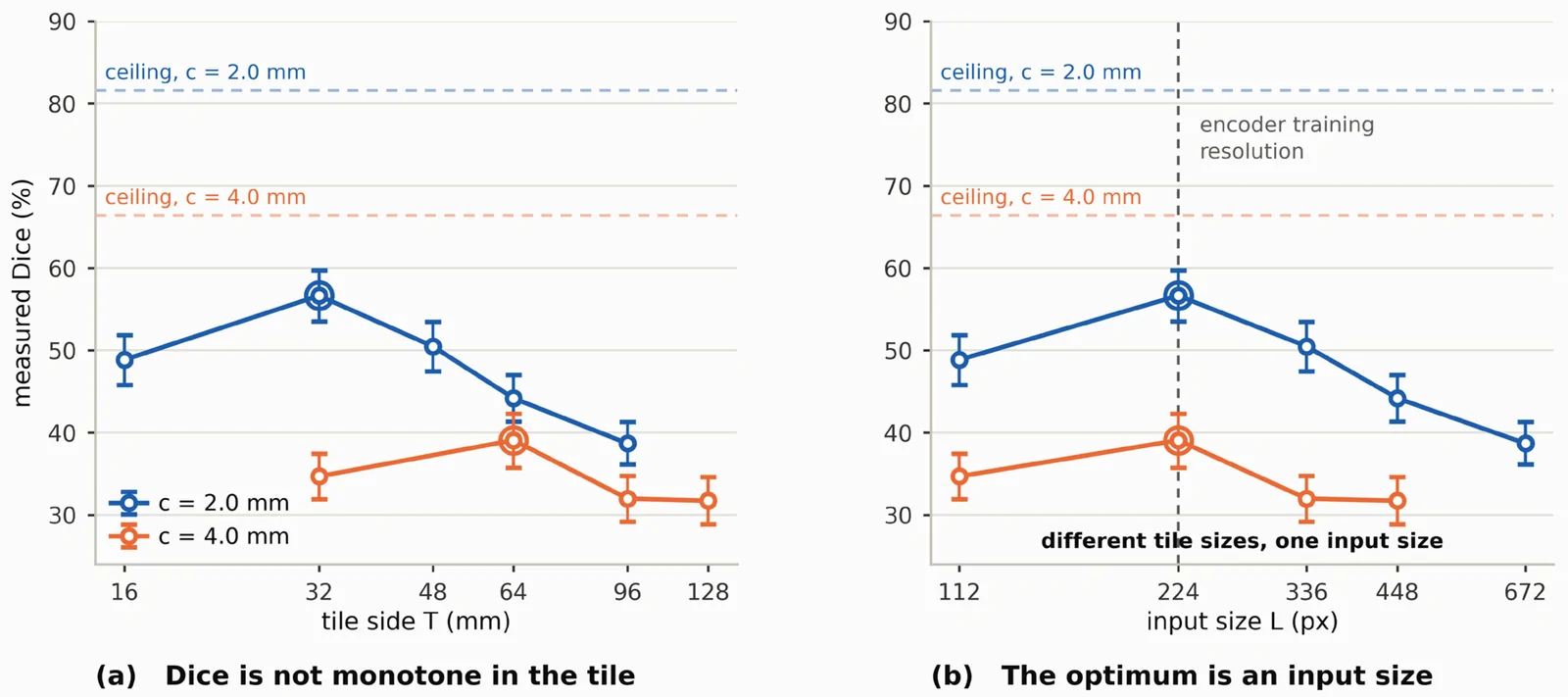
Measured Dice at a fixed feature-cell size, against the tile side and against the input resolution. (**a**) Two ladders, one at c = 2.0 mm and one at c = 4.0 mm; each holds the cell size fixed, so every point on a ladder is bounded by the dashed ceiling above it and differs only in what it attains. Both are unimodal in the tile side, and the open ring marks each maximum. (**b**) The same nine measurements against the input size. The two maxima fall at different tile sizes—32 mm and 64 mm—but at the same input size, which is the resolution the frozen encoder was trained at. Bars are cluster bootstraps over the 180 query nodules.

Dice is not monotone in the tile size. At c = 2.0 mm, it rises from 48.9% at a 16 mm tile to 56.6% at 32 mm, and then falls steadily to 38.7% at 96 mm; at c = 4.0 mm it rises from 34.7% at 32 mm to 39.1% at 64 mm and falls to 31.7% at 128 mm. Both maxima are interior, and the paired differences against the smallest tile of each ladder are significant in both directions—+7.8 points (95% CI 4.9 to 10.7) at the 32 mm tile of the first ladder and −10.2 points (−13.4 to −6.8) at its 96 mm tile. The two optima do not share a physical tile size: 32 mm in one ladder, 64 mm in the other. They share an input resolution. Both fall at L = 224 pixels, which for a ViT-B/14 is a 16 × 16 token grid and is the resolution at which the encoder was trained. Figure 12 shows both ladders.

**Figure 13:**
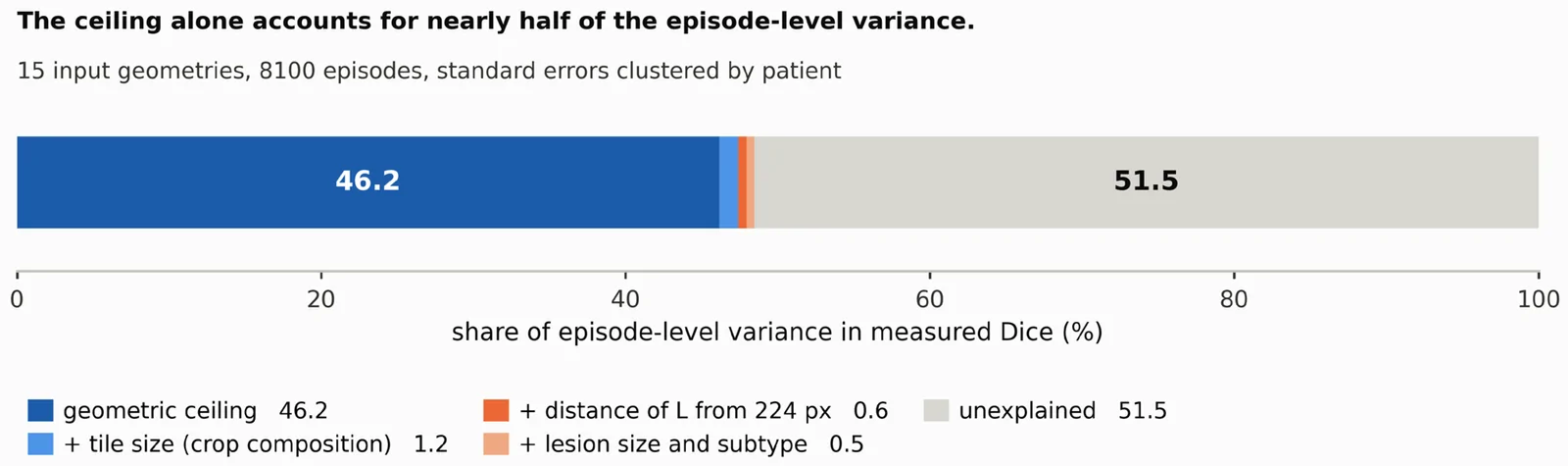
What accounts for the episode-level variance in measured Dice, over the fifteen input geometries of Table 7 and Table 8. Shares are the incremental R^2^ of nested regressions with standard errors clustered by patient, entered in the order shown. The geometric ceiling is the largest single term by an order of magnitude; the two residues that Section 3.6 separates are small beside it, and half the variance is left for the encoder, the prototype rule and the calibration.

This separates the residue of Section 3.5 into two terms rather than one. Pooling the fifteen configurations of Table 7 and Table 8—8100 episodes, standard errors clustered by patient—the ceiling alone gives R^2^ = 0.462. That figure is not comparable with the 0.540 of Section 3.5 and should not be read as a weakening. The six configurations added here carry only two distinct ceilings between them, because holding c fixed is precisely what fixes the ceiling; on those six alone the ceiling explains R^2^ = 0.187. The pooled set is therefore built to contain variation the ceiling cannot explain, and the fall from 0.540 to 0.462 measures the size of that variation rather than a loss of predictive power. Adding the logarithm of the tile side raises it to 0.474 with a negative coefficient (−0.050, *p* = 1 × 10^−6^); at equal ceiling, a smaller physical tile is better, which is the crop-composition effect. Adding the logarithm of the input size on top of that adds nothing (*p* = 0.83); input resolution has no monotone effect. Adding instead the absolute log distance of the input size from 224 pixels raises R^2^ to 0.480 with a coefficient of −0.068 (*p* = 4 × 10^−14^); moving away from the resolution the encoder was trained at costs accuracy in either direction. With both terms present, the foreground-token count is again indistinguishable from zero (*p* = 0.43). The residue is therefore a composition term that is monotone in the tile and a representation term that is not monotone in the input but roughly symmetric about the value the encoder was trained at. Neither is predicted by the geometry, and we take the second up in Section 4.1. Figure 13 gives the shares.

Under unconstrained sampling a support shares the query’s subtype in 39.1% of episodes by chance. Drawing the support from any other patient rather than from the same subtype costs 1.91 points averaged over the nine configurations, and the cost is not uniform. It is indistinguishable from zero at the two coarsest grids and largest at the finest, −3.78 points (95% CI −6.45 to −1.19) at the derived tile. The pattern is the one the geometric account predicts; where the grid already prevents the lesion from being expressed, an appearance mismatch in the support has nothing left to spoil, and where the grid permits a boundary the fidelity of the prototype begins to matter. Within the unconstrained sweep itself the same ordering appears—a support that happens to share the query’s subtype is worth 8.4 points at the derived tile and 0.1 points at the coarsest.

What does not change is the relationship this paper reports. Under unconstrained sampling, the episode-level correlation between a nodule’s ceiling and its measured Dice is 0.717 against 0.735, the correlation across configuration means is 0.971 against 0.975, the ordering of the nine configurations is identical, and the derived tile remains the best of them, attaining 64.8% of the ceiling against 69.4%. The subtype constraint is worth a few points of level and nothing of structure. We would ask a reader to take the absolute Dice of Table 7 as the optimistic end of a range whose other end is Table 9, and the attained fraction as the quantity that survives either choice.

A third sweep changes the representation rather than the geometry. Replacing the frozen DINOv2 ViT-B/14 with a frozen ImageNet-pretrained ResNet-50, taking features from the stride-16 stage, gives a convolutional extractor whose cell size can be matched to every one of the nine published configurations exactly, since c = 16 T/L admits an input size that is a multiple of 32 at each of them. Nothing else changes; the same 180 nodules, the same jitter offsets and the same 540 episode pairs. Table 10 reports it.

The convolutional extractor is weaker everywhere, and by a wide margin in the middle of the range—2.3% against 7.8% at c = 10.94 mm and 11.5% against 24.9% at c = 5.33 mm. That is a statement about the two representations and not about the bound. What the bound does is unchanged: measured Dice still rises with the ceiling across the nine configurations (r = 0.896 across configuration means, 0.655 per episode, Spearman 0.789), and the tile derived in Section 3.4 from geometry alone is again the best of the nine, attaining 60.2% of its ceiling. One pair of configurations swaps places between the two extractors—c = 10.94 mm and c = 14.00 mm, whose ceilings are 33.2% and 25.5% and whose measured values differ by 0.3 points—and we record it rather than smooth it over: the ordering is reproduced at every step where the ceilings are separated by more than a few points, and not at the one step where they are not.

### 3.7. A Trained Prototype Head at Three Cell Sizes

Every measurement reported so far is training-free, and it is reasonable to ask whether training moves the ceiling or only the distance to it. To answer it without contaminating the benchmark, we assembled a training cohort of 100 LIDC-IDRI patients disjoint from the 28 patients of the evaluation subset, selected to match its subtype composition, and containing 472 annotated nodules. On this cohort we trained a prototype head—a two-layer projection on top of the frozen DINOv2 features, with the last two transformer blocks unfrozen at a tenth of the head learning rate—with a soft-Dice and cross-entropy objective over episodes drawn in exactly the manner of Section 2.7. Training was repeated independently at three cell sizes spanning the range, and each trained model was evaluated on the same 180 nodules and the same 540 episode pairs used everywhere else in this paper, so that the trained and training-free columns of Table 11 differ in one factor only. The separation is at the patient level and was fixed before training began. The 28 evaluation patients were selected in Section 2.1 for reasons that consulted only nodule size and subtype, months before any training was contemplated; the 100 training patients were then drawn from the remainder of LIDC-IDRI with those 28 excluded by identifier, so no patient, no series and no nodule appears on both sides, and no image from an evaluation patient was seen at any point during training. Nothing measured on the evaluation cohort entered the choice of training cohort, of architecture, of learning rate or of epoch count; the three geometries were fixed in advance, each was trained once, and the run reported is that run. The trained column of Table 11 is therefore a held-out result and not a best-of selection.

Training raises measured Dice at every cell size, by 6.3 points at c = 21.88 mm, 17.4 points at c = 8.00 mm and 19.1 points at c = 2.00 mm, and it improves at least nine query nodules in ten at every geometry. What it does not do is change the shape of the curve. The trained values remain ordered by cell size in the same way; no diameter band at any of the three cell sizes exceeds its own ceiling; and the coarsest trained configuration stays far below the finest training-free one, 7.4% against 56.6%. What training buys is the distance to the bound and not the bound itself, and how much of that distance it can buy is governed by how much the geometry leaves available: the fraction of the ceiling attained rises from 52.0% at c = 21.88 mm to 75.4% at c = 8.00 mm and 92.8% at c = 2.00 mm. Where the grid cannot express the lesion there is little left for training to recover. At a single threshold held fixed across every episode the trained values are 4.9%, 30.2% and 71.6%, so neither the ordering nor the conclusion changes under the calibration a deployable system would face. This is the expected result if the bound is a property of the decision geometry rather than of the weights, and it is the reason the paper reports the bound as a constraint on what training can be asked to deliver rather than as a measurement of any particular model. The comparison also fixes the scope of the claim: training closes part of the gap between the measured value and the ceiling, and none of the gap between the ceiling and one hundred per cent.

## 4. Discussion

### 4.1. What the Bound Explains, and What It Does Not

The bound is a statement about representation geometry and nothing else. It says that if the decision is made on a grid of c millimetre cells and the prediction is a union of whole cells, then Dice cannot exceed a value fixed by ρ, whatever the features are. It does not predict the performance of any particular method, and a method that falls far below the bound has a problem the bound does not describe.

This distinction matters for how the result should be used. A reported Dice of 30% is uninformative on its own. If the ceiling at that configuration is 32%, the method is close to what its grid permits and the remedy is geometric. If the ceiling is 90%, the deficit lies in the features, the prototype construction, or the calibration, and refining the grid will not help. The quantity that separates these two cases is the ratio of the two numbers, and reporting it costs nothing once the configuration is stated. Table 7 gives it for the reference implementation across nine configurations, where it spans 7.9% to 69.4% while the method is held fixed; the same two columns would fit in any results table. We would encourage reporting both columns independently of whether the analysis presented here is adopted.

There is one respect in which the bound understates the difficulty. It grants the model a perfect cell classifier. In practice the same coarse grid that limits the output also limits the input to the prototype: when a lesion occupies less than one cell, the masked average pooling that forms the support prototype has at most one cell to average, and the prototype carries almost no lesion-specific signal. The bound is therefore optimistic by an amount that grows as ρ falls. Section 3.5 measures that amount rather than leaving it as a prediction; attainment falls from 69.4% of the ceiling at c = 2.0 mm to 7.9% at c = 21.9 mm, tracking the share of episodes whose support prototype has a single cell to average as it rises from 6% to 100%.

A reader may reasonably ask whether the derived 32 mm tile is the best of the nine configurations in Table 7 because of the geometry this paper argues for or because it happens to sit at the input resolution the encoder was trained at. Section 3.6 shows that the question is real and also bounds it. The resolution term is significant—departure from 224 pixels costs accuracy in either direction at *p* = 4 × 10^−14^—but across the fifteen configurations the ceiling alone accounts for 46.2% of episode-level variance, while the resolution term adds 0.6 points to that and the tile-composition term 1.2. The three effects are of different orders. They are also separable in this design, because the nine configurations of Table 7 vary the tile at fixed input and the input at fixed tile, while the ladders of Table 8 vary both together. The practical reading is not that the geometry was a proxy for resolution but that a configuration has to satisfy two constraints at once: Section 2.6 derives the tile at a stated input size, and the derivation is silent about whether that input size suits the backbone. For a frozen encoder it evidently does not suit it equally at every value, and a method that adapts its encoder may not carry this term at all.

### 4.2. Why Abdominal Benchmarks Could Not Have Revealed This

The curve is steep below ρ ≈ 2 and flat above ρ ≈ 6. Every target in the benchmarks this method family is evaluated on sits in the flat region: a 100 mm liver at c = 11 mm gives ρ ≈ 9, where the ceiling is above 96% and a one-cell error costs almost nothing. Two methods that differ by five points on such a target may differ by forty on a 6 mm nodule, and no amount of comparison in the flat region carries information about the steep one.

This is not a criticism of those benchmarks for what they measure. It is an argument that the field’s shared evaluation protocol has a blind spot of a specific shape, and that the blind spot is exactly where clinical interest in lung CT lies. It also implies that the ranking of methods established on abdominal organs should not be assumed to transfer, since the ranking was established where the binding constraint is absent.

For a diagnostic-imaging readership, the practical consequence is concrete, and it acts through two of the lesion groups a reader is least willing to lose. The sub-centimetre nodules that a screening study exists to find are, by their size, where the geometric ceiling is lowest; and the large solid masses that a threshold lung mask erases before segmentation even begins—six of the eighteen masses above 20 mm in our subset, an estimate whose 95% interval runs from one in six to one in two—are precisely the ones a pipeline should not be silently dropping. A method that loses accuracy on exactly these lesions while reporting a healthy pooled Dice is failing in a way a size-blind evaluation cannot see. The two remedies we give—measuring lesion size as the model sees it, and bounding the search region without erasing—are preprocessing choices rather than new models, and cost nothing to adopt.

### 4.3. Evidence from an Independent Reimplementation

A recent comparative review of few-shot medical image segmentation reimplements SSL-ALPNet and evaluates it on both abdominal organs and kidney tumours [6]. On organs it obtains Dice of 52.1 to 74.2. On one-shot kidney tumour segmentation the same implementation obtains 23.9, and the conventionally trained ALP-Net obtains 30.5. The authors attribute the drop to a mismatch between tumours and the superpixel structure the self-supervision relies on, observing that tumours “can appear as small, irregular segments within an organ.”

The observation is consistent with what we report, and it is worth being precise about how far the agreement extends. Kidney tumours are centimetre-scale, so ρ at that configuration is well above the steep part of the curve, and the ceiling alone does not account for a fall to 24%. At that scale the authors’ explanation and ours are complementary rather than competing: the grid is not the binding constraint, and something else—plausibly the self-supervision signal—is. What our result adds is that as the target shrinks toward and below the cell size, the geometric term stops being one contribution among several, and becomes the whole of the available performance. The value of separating the two is that it tells a practitioner which regime they are in, and therefore which intervention can help.

### 4.4. Four Decision Regimes, and Where the Bound Applies

The bound assumes the decision is made at grid resolution. Methods that produce their final segmentation at pixel resolution are not subject to it in this form, and the literature contains a relevant demonstration. A few-shot framework for lung nodules that uses a network’s coarse prediction only as the initial contour for a variational active-contour model, which then evolves at pixel resolution, reports Dice near 0.90 on LIDC [13]. That method is not episodic prototype matching, and the term “few-shot” refers there to the number of training patients rather than to a support–query protocol, so it is not a competitor on the same task. It is, however, direct evidence for the reading we propose: the difficulty is not that sub-centimetre nodules are intrinsically unsegmentable, but that a decision taken on a coarse lattice cannot express their boundary. It is also notable that this method operates on lung-region crops, which is the same lever, arrived at independently.

The same reasoning applies to any architecture with a learned decoder that recovers sub-cell detail, and to promptable segmenters that refine at full resolution. For those, the bound as stated here is a bound on the coarse stage only, and the interesting question becomes how much of the sub-cell detail the decoder can in fact recover—which is a measurement we do not make.

It is worth separating the four regimes explicitly, because the same number means something different in each. (i) A whole-cell grid-resolution decision, in which the prediction is a union of complete feature cells; Equation (7) is exact and cannot be exceeded at a given grid alignment. (ii) A bilinearly upsampled soft score map, which is what the prototype family actually emits; the effective partition is finer than whole cells—the region above a 0.5 threshold around one active cell is about 0.61 of that cell—and the whole-cell value is exceeded in 6.2% of our own episodes at an oracle threshold and 1.7% at a fixed one, concentrated at the finest grids. (iii) An architecture with a learned decoder that recovers sub-cell detail; Equation (7) bounds the coarse stage only, and what the decoder recovers is not measured here. (iv) A promptable or refinement method that decides at full resolution, or a variational model evolving at pixel resolution as in [13]; the bound does not apply in this form at all. SSL-ALPNet, ADNet, Q-Net and the reference implementation of Section 2.7 all sit in (ii), which is why we report the whole-cell value as a bound on their grid decision and state the relaxation beside it rather than treating the two as the same quantity.

### 4.5. Implications for How These Methods Are Reported

Three practices would make the results of this method family interpretable at lesion scale, and none of them require new experiments. A fourth follows from Section 3.6, and that one does.

The field of view of the input crop should be reported alongside the input resolution. Equation (1) shows that c is determined by both, and a paper that states only the input size has not stated the resolution at which its model decides. Two papers reporting “224 × 224 input” may be operating at cell sizes that differ by a factor of ten.

Accuracy on small targets should be reported stratified by size rather than pooled. A pooled figure on a cohort whose median lesion is 7.8 mm is dominated by whichever band is most populous, and it averages across a regime change.

Lesion size should be stated in the same terms in which the model encounters it. A two-dimensional model on an axial slice sees a cross-section whose equal-area diameter is a median 69% of the nominal diameter; papers that report two-dimensional results while describing lesion size in nominal terms are comparing quantities that differ by a third.

The input size should be reported against the backbone’s own training resolution and not only in pixels. A frozen encoder evaluated away from the resolution it was trained at loses accuracy in both directions; in the sweep of Section 3.6, moving away from 224 pixels cost accuracy at *p* = 4 × 10^−14^ regardless of which way it moved, and the two ladders reached their maxima at that input size rather than at any particular tile size. A configuration is therefore constrained twice over, once by the cell size that the crop and the network produce together and once by whether that combination lands the input near the backbone’s native size. The two constraints are independent, and a paper that reports only the first has not said enough for its result to be reproduced at another scale. We state this as a reporting practice rather than as a calibrated correction: our ladders are unbalanced in input size (Section 4.6), and a method that adapts its encoder rather than freezing it may not carry the term at all.

### 4.6. Limitations

The ceiling is measured on the largest axial slice of each nodule, in two dimensions, because that is the setting of the methods under study. Within that setting the choice of the largest slice is deliberately favourable to the methods; every other slice of the same nodule presents a smaller equal-area diameter and therefore a lower ceiling, so the per-slice bound in Table 2 is the most generous one a slice-wise pipeline could meet. A three-dimensional formulation would have a different bound, and probably a higher one, since a lesion spanning several slices covers more cells in three dimensions than the flat count suggests.

The bound is stated for predictions that are unions of whole cells. Bilinear upsampling of a soft score map relaxes it: the region above a 0.5 threshold around a single active cell is roughly 0.61 of the cell’s area, so the effective quantisation is finer than nearest-neighbour assignment implies. We report the whole-cell form because it corresponds to the decision the model actually makes, but the dependence on the upsampling operator is real and we do not conceal it. The attainment sweep uses exactly this bilinear upsampling, and there the relaxation is visible but small; measured Dice exceeds the offset-averaged whole-cell ceiling in 6.2% of episodes at an oracle threshold and 1.7% at a fixed one, and by more than a tenth in 2.5%. Two effects contribute, and the released record does not separate them. Bilinear upsampling genuinely relaxes the whole-cell form, which is why the exceedances concentrate at the finest grids—9.9% of episodes at c ≤ 4 mm against 3.3% at c ≥ 11 mm. And because Section 2.7 samples the sub-cell alignment rather than fixing it, an episode drawing a favourable alignment can beat a ceiling that averages over all of them. Neither is a violation of Equation (7), which bounds a given alignment; both are reasons to read Table 2 as a bound on the offset-averaged case, which is how Section 3.1 states it. These counts are taken against the smoothed population ceiling; a per-nodule own-mask ceiling would shift them slightly.

The lesion-preservation measurements use 180 nodules from 28 patients, a subset chosen before measurement to populate all fifteen subtype-by-diameter cells. It is adequate for the comparison it supports—the same nodules segmented twice, under six policies—and too small to characterise the LIDC population. The failure rates we report should be read as evidence that the failure mode exists and is policy-dependent, not as population estimates.

The attainment sweep uses no training, one support image per episode, and the same 180 nodules. It establishes that the ceiling bounds and orders outcomes; it does not establish what any published system would score, and its absolute Dice is lower than a trained method’s would be. The quantity we would ask a reader to carry away from Table 7 is the ratio, not the Dice. Two smaller caveats attach to it. The ceiling column reads Equation (7) off the population curve at each nodule’s ρ rather than recomputing it on that nodule’s own mask, which is justified only to the extent that the collapse onto a single curve in Section 3.1 is exact; 1.0% of episodes fall outside the tabulated range of ρ and take the nearest tabulated value, which moves the pooled attainment by half a point. And the two configurations sharing c = 2.0 mm attain different fractions of the same ceiling, so tile size carries information that cell size does not. The procedure of Section 2.6 fixes c and is silent on that residue. Section 3.6 now quantifies it: at fixed c, the outcome is unimodal in the tile size with an interior optimum, and the residue separates into a monotone crop-composition term and a non-monotone term that penalises departure from the encoder’s training resolution. The procedure should therefore be read as fixing the ceiling rather than the outcome. Section 3.6 adds a second frozen representation, and Section 3.7 trains a prototype head on a cohort of 100 patients disjointed from the evaluation subset. Training raises the measured value at every cell size without reordering the configurations, which is what the bound predicts. What is still not measured here is a fully end-to-end trained encoder of the kind the named methods use, or a training cohort large enough to saturate that training; both are the subject of the companion study.

The tile evaluation is candidate-centred, and a deployable pipeline is not. Query tiles are placed on the consensus-mask centroid with the ±8 mm offset of Section 2.7, which samples the sub-cell alignment and stands in for localisation error; a real system must first produce the candidate position, and the error distribution of an actual nodule detector is neither Gaussian nor bounded at 8 mm. Everything this paper says about the 32–42 mm window is therefore conditional on a candidate generator accurate to roughly that tolerance. Measuring the ceiling under a detector’s own error distribution, rather than under a uniform perturbation, is the natural next step and is not taken here.

Support and query are paired within nodule subtype, which is favourable. In an operational few-shot setting the subtype of the query is what one is often trying to establish, and an unconstrained support draw would place a solid support against a ground-glass query in a share of episodes. We adopt the constrained pairing because it isolates geometry from appearance mismatch, which is the comparison the paper is making, but it does mean the absolute Dice of Table 7 is an optimistic reading of the same implementation under unconstrained sampling. The size of that optimism is now measured. Section 3.6 repeats the nine configurations with the support drawn from any other patient and finds a cost of 1.91 points averaged over them and 3.78 points at the derived tile, with the episode-level correlation, the ordering of the configurations and the identity of the best one unchanged. The practical reading is therefore the following. Same-subtype pairing corresponds to a deployment in which the support example is chosen by a reader who already knows what the query looks like, which is not the situation few-shot segmentation is proposed for; routine use is closer to the unconstrained draw, where subtype is among the things one is trying to establish. Table 9 quantifies the difference so that the reader does not have to assume it: 1.91 points averaged over the nine configurations and 3.78 points at the derived tile, against a ceiling of 81.6% at that configuration. The absolute Dice we report should therefore be read as the optimistic end of a range whose other end is Table 9, while the attained fraction, the ordering of the configurations and the identity of the best one are the same under either choice. We state the constraint here rather than removing it because the paired design is what makes the geometric comparison clean; the unconstrained sweep is what makes it applicable.

The two ladders of Section 3.6 are unbalanced in input size. Across the fifteen configurations L = 224 appears five times, L = 336 four, L = 448 three, L = 112 twice and L = 672 once, so the resolution term is carried by few configurations at its extremes and its coefficient should be read as an indication of sign and order of magnitude, rather than as a calibrated penalty. The ladders also confound tile size with input size by construction—holding c fixed forces L = 14 T/c—so the separation of the two terms rests on the nine configurations of Table 7, where they do vary independently. A design that varied the input size at a fixed tile across more values would estimate the resolution term better, and would also say whether the optimum sits exactly at 224 pixels or merely near it. We did not run one, because the claim we need is only that a second, non-geometric term exists and is small beside the ceiling.

Acquisition heterogeneity is not analysed. LIDC-IDRI was assembled across seven academic centres and eight companies, and spans a wide range of scanner manufacturers, reconstruction kernels and slice thicknesses; the subset used for the pixel-data measurements is restricted to series at or below 2.5 mm slice thickness, but is not balanced across the other axes. Reconstruction kernel in particular affects the texture a frozen encoder sees, and could shift representation quality independently of the geometry this paper isolates. The ceiling itself is a property of the mask and is unaffected; attainment is not. Three parameters are worth naming because they act through different parts of the chain. Reconstruction kernel changes the texture a frozen encoder sees without changing the mask, so it can move attainment while leaving the ceiling exactly where it is; a sharp kernel and a smooth one presented at the same cell size are bounded identically and need not score identically. Slice thickness acts earlier and differently: it sets how much of a lesion falls in the slice a two-dimensional model is shown, and therefore moves the slice-equivalent diameter and with it ρ, which is the variable the ceiling depends on. Restricting to series at or below 2.5 mm bounds that effect, but does not remove it. The scanner vendor and tube current act mainly through noise, which again is an attainment term. Even within our restricted subset, the acquisition spread is not small—in-plane pixel spacing runs from 0.53 to 0.95 mm across the 28 series, a factor of 1.8—and we did not stratify by it, because 28 series cannot support a factorial analysis of vendor, kernel and thickness together. A study that varied these deliberately at a fixed cell size would separate the representation term from the geometric one more sharply than we can here, and we would expect it to move the measured values and leave the bound and the ordering intact.

All measurements come from a single collection. LIDC-IDRI is the only public lung CT corpus with multi-reader lesion contours at this scale, which is why it is used, but the ρ-to-ceiling relationship has not been confirmed on a cohort annotated under a different protocol. The geometric part of the argument is protocol-independent in the sense that Equation (7) is a statement about any binary mask; what an external cohort would test is whether the lesion size distribution, and therefore where these methods sit on the curve, transfers. We would expect the shape of the curve to transfer, and the operating point not necessarily.

Finally, calling the crop the cheapest lever conflates two costs that should be kept apart. It is the cheapest in parameters: no architectural change, no additional weights, no training. It is not the cheapest in computation. Covering a lung region of roughly 280 × 280 mm in-plane with 32 mm tiles at 50% overlap takes 17 × 17 = 289 tiles per axial slice against a single forward pass for a full-chest input of the same slice—72,250 encoder evaluations for a 250-slice volume—and 81 per slice if the tiles are not overlapped at all. A 42 mm tile at the same overlap takes 169. Exhaustive tiling is therefore not the intended deployment: the tile is meant to be placed on candidates supplied by a detector, which reduces the count to a few passes per candidate and is the same assumption the previous paragraphs identify as load-bearing. Wall-clock time and peak GPU memory depend on the accelerator and the batching, and are not measured here; the tile counts above are exact and are what the comparison should be made on.

Finally, the bound is a property of a deployed configuration and not of prototype-based segmentation as an idea. The same architecture evaluated at a larger input, a smaller crop, or a finer stride moves along the curve. We describe the ceiling as a property of how these methods are commonly deployed, and we would regard a paper that deployed them at ρ > 6 on nodules as having taken the point.

### 4.7. Summary in Plain Terms

A segmentation network does not decide pixel by pixel. It decides on a coarse internal grid and then enlarges the answer to the size of the image. On a whole-chest CT resized to a typical network input, one square of that internal grid covers about two centimetres of the patient. Most lung nodules are smaller than one centimetre. The lesion is therefore smaller than the smallest region about which the network can make a separate decision, and no amount of training recovers a boundary the network has no way to express. That is the whole of the argument; the rest of this paper measures how large the effect is and what can be done about it.

Two consequences follow for practice, and neither needs a new model. First, the size that governs performance is the size of the lesion on the one slice the network is shown, not the diameter recorded in the report: the former is typically about two thirds of the latter, so a nodule described as 9 mm behaves like a 6 mm one. Second, showing the network a smaller region of the chest shrinks the grid square in proportion, and this is the cheapest improvement available. But the region must be selected by cropping, not by blanking out everything outside an automatic lung outline. Such an outline cuts through lesions that touch the chest wall or the mediastinum, and in the subset examined here it removed more than half the area of six of the eighteen masses larger than 2 cm—the lesions a reader would least accept losing. Cropping to the same region without altering the pixel values costs one morphological operation and loses nothing.

What this study does not do is measure how well any published method performs. It measures how well any method could perform at a given input geometry, and how much of that a deliberately simple, untrained implementation recovers. Placing a trained system against the same bound is the subject of the companion study.

## 5. Conclusions

Prototype-based few-shot segmentation does not fail on pulmonary nodules only because lung tissue is difficult. It fails, at the input configurations it is commonly deployed with, in part because the lesion is smaller than the cell on which the decision is taken—a term that is fixed before any feature is computed and that dominates the others as ρ falls. This is not a demonstration that tissue contrast, feature quality, self-supervised pretraining, prototype construction, calibration or domain shift are irrelevant in trained systems; those factors govern how much of the bound a system recovers, and the present study does not measure them. We quantified this by measuring, on the 50% consensus masks of 2614 LIDC-IDRI nodules, the best Dice any classifier deciding at a given feature cell grid could achieve, and showed that the result collapses onto a single curve (R^2^ = 0.996) in the ratio of lesion diameter to cell size. At the configuration reported for SSL-ALPNet the bound on the most populous diameter band is 19.0%, against the 63–85% the same method reports on abdominal organs.

Two corrections follow for how such results should be stated. The governing size is the equal-area diameter of the slice the model sees, not the nominal three-dimensional diameter, and using the latter overstates the bound by 11.5 points. The cell size is set as much by the field of view of the crop as by the network, so the crop is a design variable rather than a preprocessing detail.

Acting on the second correction requires care. Restricting the search region to the lung is the cheapest way to reduce the cell size, but a lung mask used to erase discards part of the lesion it was meant to help find: hard masking removes more than half of 11.7% of nodules, and a learned mask reduces this to 2.2% without eliminating it. The remedy is not a better mask but a different use of it. A mask that bounds the search region without altering voxel intensities preserves every lesion we measured while delivering the same reduction in cell size. How far to reduce it also follows from measurement rather than from a chosen margin: inverting the ceiling fixes the tile from above, truncation measured on every mask fixes it from below, and at a 224-pixel input the two leave a window of 32 to 42 mm.

How close a real system comes to the bound is method-dependent rather than geometric, but it is not beyond measurement. Swept across nine input geometries with no training of any kind, a reference implementation of the same prototype rule attains 69% of the ceiling at the derived 32 mm tile and 8% of it at a full-chest input, and its measured Dice tracks the ceiling at r = 0.74 per episode and 0.98 across configuration means; a quantity computed from consensus masks alone, with no model in the loop, orders the outcomes of a model. The gap that remains is widest exactly where the grid also starves the support prototype, which is the second cost of a coarse cell and one the bound does not charge for. Whether trained methods recover more of the bound than frozen representations do is the subject of a companion study. The 32–42 mm window this study derives is conditional in the same way: it holds for the target ceiling and the 25th-percentile diameter chosen in Section 2.6, and it presumes a candidate position accurate to about the ±8 mm the sweep perturbs by. Holding the cell size fixed and moving the tile reveals a further residue the geometry does not predict either: the outcome is unimodal in tile size and peaks at the resolution the frozen encoder was trained at, falling away on both sides of it. Relaxing the same-subtype pairing costs a few points of level and leaves the ordering intact.

## Figures and Tables

**Table 1:** Input geometry of the prototype-based methods this paper reasons about.

Method	Backbone, s (px)	L (px)	FOV (mm)	c (mm)	Target	Size (mm)	Decision
SSL -ALPNet [1]	ResNet -101, 8	256	350 †	11.0	abdomen	50–150	grid
DINOv2-ALPNet [5]	ViT-L/14, 14	672	350 †	7.3	abdomen	50–150	grid
MSPO- Net [10]	VGG-16, 8	417	not stated	—	lung	10–50	grid
Active contour [13]	—	—	lung crop	—	nodules	3–30	pixel
This work, full chest	ViT-B/14, 14	224	350	21.9	nodules	3–35	grid
This work, 32 mm tile	ViT-B/14, 14	224	32	2.0	nodules	3–35	grid

The feature-cell size c follows from Equation (1) as s FOV/L, where s is the patch size of a vision transformer or the output stride of a convolutional backbone, in pixels. Decision records where the final label is produced; grid means on the feature grid and then bilinearly upsampled, pixel means at full resolution. The bound of Equation (7) applies exactly to whole-cell decisions, is relaxed by bilinear upsampling, and does not apply at pixel resolution. † The source does not state the physical field of view of its input; 350 mm, the nominal chest field of view used throughout this paper, is assumed. Three further prototype methods evaluated on the same abdominal benchmarks [2,3,4] state neither the field of view nor, in two cases, the input size, and cannot be placed in this table at all.

**Table 3:** The two size definitions and the ceiling each predicts, against the measured value.

Band	Nominal d (mm)	Slice-Equivalent d (mm)	Ceiling From Nominal	Ceiling From Slice Area	Measured
3–5	4.50	2.90	19.1	9.2	9.4
5–8	6.43	4.39	32.1	18.5	19.0
8–10	8.90	6.16	46.8	30.4	30.1
10–20	13.43	9.56	62.5	48.6	46.6
20–35	26.11	17.83	79.4	70.2	68.2

At c = 11 mm. Diameters are band means over the 2614 nodules. Measured is the mean oracle Dice of Equation (7) computed on each nodule’s own consensus mask at that cell size, averaged over the band and over the 6 × 6 offset lattice of Equation (8); it is the quantity the two ceiling columns are predictions of, and it is not the output of any segmentation model.

**Table 4:** Nodules losing more than half of their area, by mask-handling policy.

Policy	Threshold Mask	Learned Mask (R231)
hard masking (erase outside)	**11.7%**	**2.2%**
morphological closing, then erase	2.2%	0.6%
convex-hull fill, then erase	0%	0%
dilate 5 mm, then erase	0%	0%
dilate 10 mm, then erase	0%	0%
dilate 15 mm, then erase	0%	0%
crop to bounding box, no erasing	0% by construction	0% by construction

*n* = 180 nodules from 28 patients. The two hard-masking rates carry wide intervals at this sample size: threshold mask 11.7% (95% CI 7.8–17.2%) and learned mask 2.2% (0.9–5.6%), Wilson. Bold marks the hard-masking rates, the only policy that leaves a failure tail.

**Table 5:** Nodules losing more than half under hard masking, by nominal diameter band. Bold marks the band in which the failure concentrates.

Band	*n*	Threshold Mask	Learned Mask
3–5 mm	22	9.1%	0.0%
5–8 mm	60	10.0%	1.7%
8–10 mm	37	13.5%	2.7%
10–20 mm	43	4.7%	2.3%
**20–35 mm**	18	**33.3%**	5.6%

**Table 6:** The ρ a target ceiling requires, and the tile size it implies.

Target Ceiling	50%	60%	70%	75%	80%	85%
required ρ*	0.86	1.10	1.55	1.91	2.37	3.22
required c* (mm)	4.73	3.70	2.63	2.13	1.72	1.26
T* at L = 224 (mm)	76	59	42	34	27	20
T* at L = 336 (mm)	114	89	63	51	41	30
T* at L = 448 (mm)	151	118	84	68	55	40

For a ViT/14 backbone at three input sizes, guaranteeing the target for the smallest 25% of LIDC nodules, whose slice-equivalent diameter is 4.07 mm. Section 2.6 gives the reason for that quantile and for the target range.

**Table 7:** Measured Dice of the training-free reference implementation against the ceiling.

c (mm)	T (mm)	L (px)	Measured	95% CI	Fixed Thr.	Ceiling	Attained
21.88 ^d^	350	224	1.1	0.9–1.4	0.4	14.3	7.9
14.58	350	336	3.7	2.9–4.6	0.8	24.4	15.3
14.00	224	224	4.3	3.5–5.3	1.7	25.5	16.9
10.94 ^c^	350	448	7.8	6.2–9.4	1.3	33.2	23.4
8.00	128	224	15.8	13.6–18.1	9.0	44.0	35.9
5.33	128	336	24.9	22.2–27.6	14.2	57.8	43.0
4.00	64	224	39.1	35.9–42.3	23.3	66.4	58.9
2.00	64	448	44.2	41.4–46.9	27.6	81.6	54.2
**2.00** ^a^	**32**	**224**	**56.6**	53.5–59.7	36.2	**81.6**	**69.4**

All values are percentages, over 540 paired episodes per configuration. Superscripts follow Table 2: ^a^ is the 32 mm tile derived in Section 3.4, ^c^ SSL-ALPNet, ResNet-101 at output stride 8, 256-pixel input and ^d^ the ViT-B/14 full-chest input at 224 pixels. Bold marks the derived 32 mm tile. Measured is the best of forty per-episode thresholds, which bounds calibration out of the comparison; fixed thr. is the Dice at one threshold held constant across every episode of a configuration, selected once rather than per episode, and is the number a deployable system would face. Confidence intervals are cluster bootstraps over the 180 query nodules. Attained is the ratio of the two column means and is computed from the oracle-threshold column.

**Table 8:** Measured Dice at a fixed feature-cell size as the tile side changes.

c (mm)	T/L (mm/px)	Measured	95% CI	Fixed Thr.	Ceiling	Attained
2.00	16/112	48.9	45.8–51.9	32.8	81.6	59.9
2.00	32/224	56.6	53.5–59.7	36.2	81.6	69.4
2.00	48/336	50.5	47.5–53.5	32.3	81.6	61.9
2.00	64/448	44.2	41.4–47.0	27.6	81.6	54.2
2.00	96/672	38.7	36.1–41.3	21.0	81.6	47.4
4.00	32/112	34.7	31.9–37.5	17.8	66.4	52.3
4.00	64/224	39.1	35.7–42.3	23.3	66.4	58.9
4.00	96/336	32.0	29.2–34.7	19.9	66.4	48.2
4.00	128/448	31.7	28.9–34.6	16.2	66.4	47.8

The ceiling depends on c alone and is therefore constant within each block, so every row of a block is bounded by the same value and differs only in what it attains. Holding c fixed forces L = 14 T/c, so the tile side and the input size move together; the token grid is L/14 per axis, that is, 8, 16, 24, 32 and 48. Confidence intervals are cluster bootstraps over the 180 query nodules. The rows at T = 32/L = 224 and T = 64/L = 448 are the same as those in Table 7, and reproduce them exactly.

**Table 9:** The cost of drawing the support without regard to subtype.

c (mm)	T/L (mm/px)	Same Subtype	Unconstrained	Difference	95% CI
21.88	350/224	1.1	1.3	+0.15	−0.08 to +0.40
14.58	350/336	3.7	2.9	−0.82	−1.58 to −0.11
14.00	224/224	4.3	4.0	−0.28	−1.01 to +0.47
10.94	350/448	7.8	6.6	−1.14	−2.22 to −0.06
8.00	128/224	15.8	13.7	−2.09	−3.56 to −0.72
5.33	128/336	24.9	22.0	−2.88	−4.61 to −1.21
4.00	64/224	39.1	35.7	−3.42	−5.61 to −1.30
2.00	32/224	56.6	52.9	−3.78	−6.45 to −1.19
2.00	64/448	44.2	41.3	−2.88	−4.98 to −0.89

At the nine configurations of Table 7, differences are paired by query nodule and their intervals are cluster bootstraps over the 180 queries.

**Table 10:** The same sweep with a convolutional extractor in place of the vision transformer.

c (mm)	T (mm)	DINOv2	ResNet-50	Ceiling	Attained, DINOv2	Attained, ResNet
21.88	350	1.1	1.0	14.3	7.9	7.1
14.58	350	3.7	1.9	24.4	15.3	7.6
14.00	224	4.3	2.6	25.5	16.9	10.2
10.94	350	7.8	2.3	33.2	23.4	7.0
8.00	128	15.8	6.5	44.0	35.9	14.8
5.33	128	24.9	11.5	57.8	43.0	19.8
4.00	64	39.1	24.6	66.4	58.9	37.1
2.00	64	44.2	27.6	81.6	54.2	33.9
2.00	32	56.6	49.1	81.6	69.4	60.2

All values are percentages over the same 540 paired episodes per configuration. The convolutional extractor is an ImageNet-pretrained ResNet-50 taken at its stride-16 stage and frozen; the input size is chosen so that c matches the transformer configuration on the same row to two decimal places.

**Table 11:** The training-free reference implementation and a trained prototype head, at three cell sizes.

c (mm)	T/L (mm/px)	Training-Free	Trained	Fixed Thr.	Ceiling	Attained, Trained
21.88	350/224	1.1	7.4	4.9	14.3	52.0
8.00	128/224	15.8	33.2	30.2	44.0	75.4
2.00	32/224	56.6	75.7	71.6	81.6	92.8

All values are percentages over the same 540 paired episodes per configuration. The training-free column repeats the corresponding rows of Table 10. The trained column is the mean over the same episodes after training on 472 nodules from 100 patients disjointed from the evaluation subset; ceiling is the oracle bound of Equation (7), which does not depend on the model and is therefore identical in both cases. Cluster bootstraps over the 180 query nodules give 95% intervals of 6.0–8.9, 30.3–36.1 and 73.1–78.2 for the three trained values.

## Data Availability

This study analysed only publicly available data and created no new patient data. All image data are from the LIDC-IDRI collection of The Cancer Imaging Archive, Version 4 (updated 21 September 2020), https://doi.org/10.7937/K9/TCIA.2015.LO9QL9SX, accessed on 9 July 2026, used in compliance with its Creative Commons Attribution 3.0 licence and with the TCIA Data Usage Policy; the required attribution is given by the data citation [23] and by references [22,24], and no attempt was made to identify or contact individual participants. Annotations were read with pylidc 0.2.3 (MIT licence) [25], which distributes them with the package. The derived tables and the code that produces every table and figure in this article are archived at Zenodo, https://doi.org/10.5281/zenodo.21721043, under the MIT licence for the code and CC BY 4.0 for the derived data. The per-episode record behind Section 3.5 and Figure 11 has been released in full—4860 rows carrying the configuration, the query and support identifiers, the nominal and slice-equivalent diameter, the number of support cells inside the lesion, and Dice at both the oracle and the fixed threshold—so that the attainment column can be recomputed under a different definition of the ceiling. The corresponding record for a run with the tile centred exactly, which fixes the sub-cell alignment at its least favourable value instead of sampling it, is released alongside it. The ceiling analysis of Section 3.1 requires no pixel data and is reproducible from the released code and the pylidc package alone.
